# APP Is a Context-Sensitive Regulator of the Hippocampal Presynaptic Active Zone

**DOI:** 10.1371/journal.pcbi.1004832

**Published:** 2016-04-19

**Authors:** Melanie Laßek, Jens Weingarten, Martin Wegner, Benjamin F. Mueller, Marion Rohmer, Dominic Baeumlisberger, Tabiwang N. Arrey, Meike Hick, Jörg Ackermann, Amparo Acker-Palmer, Ina Koch, Ulrike Müller, Michael Karas, Walter Volknandt

**Affiliations:** 1 Institute for Cell Biology and Neuroscience, Biologicum, Johann Wolfgang Goethe-University, Frankfurt am Main, Germany; 2 Institute for Molecular Bioinformatics, Johann Wolfgang Goethe-University, Frankfurt am Main, Germany; 3 Institute of Pharmaceutical Chemistry, Cluster of Excellence “Macromolecular Complexes”, Johann Wolfgang Goethe-University, Frankfurt am Main, Germany; 4 SunChrom Wissenschaftliche Geräte GmbH, Friedrichsdorf, Germany; 5 Thermo Fisher Scientific, Bremen, Germany; 6 Department of Pharmacy and Molecular Biotechnology, University Heidelberg, Heidelberg Germany; University of California San Diego, UNITED STATES

## Abstract

The hallmarks of Alzheimer’s disease (AD) are characterized by cognitive decline and behavioral changes. The most prominent brain region affected by the progression of AD is the hippocampal formation. The pathogenesis involves a successive loss of hippocampal neurons accompanied by a decline in learning and memory consolidation mainly attributed to an accumulation of senile plaques. The amyloid precursor protein (APP) has been identified as precursor of Aβ-peptides, the main constituents of senile plaques. Until now, little is known about the physiological function of APP within the central nervous system. The allocation of APP to the proteome of the highly dynamic presynaptic active zone (PAZ) highlights APP as a yet unknown player in neuronal communication and signaling. In this study, we analyze the impact of APP deletion on the hippocampal PAZ proteome. The native hippocampal PAZ derived from APP mouse mutants (APP-KOs and NexCreAPP/APLP2-cDKOs) was isolated by subcellular fractionation and immunopurification. Subsequently, an isobaric labeling was performed using TMT^6^ for protein identification and quantification by high-resolution mass spectrometry. We combine bioinformatics tools and biochemical approaches to address the proteomics dataset and to understand the role of individual proteins. The impact of APP deletion on the hippocampal PAZ proteome was visualized by creating protein-protein interaction (PPI) networks that incorporated APP into the synaptic vesicle cycle, cytoskeletal organization, and calcium-homeostasis. The combination of subcellular fractionation, immunopurification, proteomic analysis, and bioinformatics allowed us to identify APP as structural and functional regulator in a context-sensitive manner within the hippocampal active zone network.

## Author Summary

More than 20 years ago, the amyloid precursor protein (APP) was identified as the precursor protein of the Aβ peptide, the main component of senile plaques in brains affected by Alzheimer’s disease. However, little is known about the physiological function of amyloid precursor protein. Allocating APP to the proteome of the structurally and functionally dynamic presynaptic active zone highlights APP as a hitherto unknown player within the presynaptic network. The hippocampus is the most prominent brain region for learning and memory consolidation, and a vulnerable target for neurodegenerative disease, e. g. Alzheimer’s disease. Therefore, our experimental design is focused on the hippocampal neurotransmitter release site. Currently, the underlying mechanism of how APP acts within presynaptic networks is still elusive. Within the scope of this research article, we constructed a network of APP within the presynaptic active zone and how deletion of APP affects these individual networks. We combine bioinformatics tools and biochemical approaches to address the dataset provided by proteomics. Furthermore, we could unravel that APP executes regulatory functions within the synaptic vesicle cycle, cytoskeletal rearrangements and Ca^2+^-homeostasis. Taken together, our findings offer a new perspective on the physiological function of APP in the central nervous system and may provide a molecular link to the pathogenesis of Alzheimer’s disease.

## Introduction

Alzheimer’s disease (AD), characterized by a massive loss of synapses, cognitive decline and behavioral changes, is mainly associated with an accumulation of neurofibrillary tangles and senile plaques [1–3]. The most prominent brain region affected by the progression of AD is the hippocampal formation. The pathogenesis involves a successive loss of hippocampal neurons accompanied by a decline in learning and memory consolidation. More than 20 years ago, the amyloid precursor protein (APP) was cloned and identified as precursor of Aβ-peptides, the main constituents of senile plaques [4,5]. Within the last decades much effort has gone into understanding the pathogenesis of AD. However, little is known about the physiological role of APP within the central nervous system (CNS). Currently, a variety of functions have been proposed, including neurite outgrowth, synaptogenesis, and synaptic plasticity, but the underlying molecular mechanism by which APP executes its functions in neurons is still elusive [6–10]. Allocating APP to the proteome of the presynaptic active zone (PAZ), a highly dynamic substructure of the presynapse, identifies APP as an yet unknown player within the neuronal communication and signaling network [11]. The presynaptic active zone is the central setting, where synaptic vesicles release their neurotransmitter into the synaptic cleft, after the arrival of an action potential and the calcium-triggered docking and fusion process [12,13]. Neuronal communication and signal transduction is highly dependent on the concerted action of individual proteins within the PAZ [14]. The multitude of individual proteins identified by proteomic analysis at hippocampal neurotransmitter release sites [14] emphasizes the need for identification and characterization of functional protein clusters involved in the regulation of neurotransmitter release. Within this study we combined state-of-the-art proteomics with bioinformatics to analyze the impact of APP deletion on the proteome of the hippocampal presynaptic active zone. We compared the PAZ proteomes of wild type controls, single APP-KO mice, and conditional APP/APLP2 double knockout mice for creating a common core proteome of hippocampal neurotransmitter release sites. Lack of APP induces only a mild phenotype in young animals (e. g. reduced body weight, reduced grip strength) [10]. This condition is changed in the elderly mice, when impairments in spatial and working memory become obvious [7,9,10]. Compared to the APP-KO mice, deletion of both APP and APLP2 provokes a lethal phenotype [15]. To circumvent postnatal lethality a conditional APP/APLP2 double knockout has recently been designed making use of the NexCre mouse line [7]. Within excitatory forebrain neurons Cre recombinase becomes activated under the control of NEX at embryonic day E11.5 allowing deletion of floxed proteins in distinct brain regions such as the hippocampus [16]. The NexCre APP/APLP2 cDKO (NexCre-cDKO) turned out to be viable but displayed severe impairments in memory related tasks associated with reduced long term potentiation (LTP) already in young animals [7]. The effect of APP deletion on hippocampus-specific function (LTP) points to an essential role of APP for learning and memory consolidation. Furthermore, the impact of APP deletion and APP/APLP2 deletion within the CNS point to general physiological role in synaptic plasticity and synaptic maintenances [6–10]. In this context, we addressed the question of the cellular function of APP at synaptic release sites by comparing wild type and knockout mice and creating protein-protein interaction networks. Former observations point to an as yet unknown link that incorporates APP into the physiological network of the presynaptic active zone. Our novel experimental design for the isolation and purification of the native hippocampal PAZ [14] along with quantitative biochemical and proteomic approaches allowed us to compare APP-mutants to their controls and to unravel the proximate impact of APP deletion. Apart from this methodological approach we visualized the impact of APP on the hippocampal PAZ proteome by creating protein-protein interaction (PPI) networks that incorporate APP into protein networks of the synaptic vesicle cycle, cytoskeletal organization and calcium-homeostasis. Or results derived from the combination of multidisciplinary biological techniques suggest that APP is as structural and functional regulator in a context-sensitive manner within the hippocampal active zone network.

## Materials and Methods

### Animals

Animal treatment was performed under veterinary supervision in accordance with animal welfare regulations of the German animal protection law (Regierungspräsidium Darmstadt and Karlsruhe, Germany). All mice were maintained in an animal facility, and the experimental procedures were approved by the Animal Welfare office of the Regierungspräsidium Darmstadt and Karlsruhe. APP-KO (APP^-/-^) compared to wildtype (C57BL/6N), and APP^flox/flox^APLP2^−/−^NexCre^+/T^ (NexCre-cDKO) compared to APP^flox/flox^APLP2^-/-^ (APLP2^-/-^), mice of both sexes and age-matched (12 or 16 weeks respectively) were kept under 12 h light and dark cycle with food and water ad libitum. The generation of APP mutant mice has previously been described [7,17].

### Antibodies

Antibodies were directed against APLP1 (CT-11, rabbit polyclonal, 1:2000, Calbiochem—Merck Millipore, Darmstadt, Germany) APP (Y188, rabbit monoclonal, 1:1000, Abcam, Cambridge, UK), Munc18 (rabbit polyclonal, 1:1000 Synaptic Systems, Göttingen, Germany), NCAM (rabbit polyclonal, 1:1000, Abcam, Cambridge, UK), SNAP25 (mouse monoclonal 1:1000 Synaptic Systems, Göttingen, Germany), synaptophysin (G63, rabbit polyclonal, 1:1000, kindly donated by Dr. R. Jahn, Göttingen, Germany), synaptotagmin-1 (rabbit polyclonal, 1:1000, Synaptic System, Göttingen, Germany), syntaxin-1 (mouse monoclonal, 1:1000 Synaptic Systems, Göttingen, Germany), SV2 (the clone CKK 10H4 producing the monoclonal anti-SV2 antibody, kindly donated by Dr. Regis B. Kelly, San Francisco, CA, USA; was cultured in-house), SV2A (rabbit polyclonal, 1:1000 kindly donated by Dr. S. Bajjalieh, Seattle, USA), and VAMP2 (cl. 69.1, mouse monoclonal, 1:1000 Synaptic Systems, Göttingen, Germany).

Dynabeads M-280 conjugated with monoclonal sheep anti-mouse IgGs (cat. No. 112.02D) were purchased from Invitrogen, Darmstadt, Germany.

### Subcellular fractionation of the PAZ from mouse hippocampus

The hippocampus was dissected from native mouse brain prior to subcellular fractionation. Synaptic vesicles were isolated from synaptosomes according to the protocol guidelines of Whittaker [18]. The protocol has previously been adapted to the fractionation of individual mouse brains [19] and downscaled for individual mouse brain regions [14]. The following modifications were applied: Individual hippocampi was homogenized in 0.4 mL of preparation buffer (5 mM Tris-HCl, 320 mM sucrose, pH 7.4) containing the protease inhibitors antipain, leupeptin, chymostatin (2 μg/mL each), pepstatin (1 μg/mL) and benzamidine (1 mM). Unless otherwise mentioned the material was kept at 4°C during the entire preparation. The hippocampal homogenate was centrifuged using a Beckman TLX Optima 120 and rotor TLA 120.2 by acceleration (mode 4) up to 2800_gav_ for 2 min. The resulting pellet was discarded and the supernatant was further fractionated by discontinuous Percoll gradient centrifugation. The Percoll gradient was prepared by layering 1.0 mL supernatant solution onto three layers of 1.0 mL Percoll solution [3%, 10%, 23% (v/v) in preparation buffer]. After centrifugation using the TLA 100.4 rotor for 7 min at 35,000_gav_, fractions containing synaptosomes were collected and diluted twofold in preparation buffer and centrifuged using TLA 100.4 rotor for 35 min at 50,000_gav_. For hypoosmotic lysis of synaptosomes the resulting pellet was triturated in four volumes of lysis buffer (5 mM Tris-HCl, pH 7.4) at room temperature. The suspension was centrifuged using the TLA 100.4 rotor for 60 min at 250,000_gav_. The pellet was resuspended and homogenized in 300 μL sucrose buffer (10 mM HEPES-NaOH, 0.5 mM EGTA, 0.1 mM MgCl_2_, 200 mM sucrose, pH 7.4). This microsomal solution was layered onto 900 μL of a discontinuous sucrose gradient (0.3 M, 0.75 M, and 1.2 M; containing 10 mM HEPES, 0.5 mM EGTA, adjusted to pH 7.4) and centrifuged using a WX Ultra 90 Sorvall centrifuge and the TST 55.5 rotor for 2 h at 65,000_gav_. Thirty-six fractions (35 μL each) were collected from top to bottom of the gradient. The pooled lower fractions (LF) 16 to 30 corresponding to sucrose concentrations of 0.5 to 1.1 M were further analyzed.

Immunopurification of the hippocampal presynaptic active zone via docked synaptic vesicles

The immunopurification protocol for the presynaptic active zone (PAZ) via docked synaptic vesicles was as described recently for individual mouse brain regions [14]. In brief, 100 μL magnetic beads pre-coupled with an anti-mouse monoclonal antibody were washed with Tris-buffered saline (TBS, pH 7.4) and incubated with TBS containing 1% glycine, 1% lysine and 0.5% saponin followed by three washing steps in TBS. Magnetic beads were then incubated for 1 h with the anti-SV2 antibody (3 μg of antibody per 107 magnetic beads to gain representative SV2 population). Crosslinking of the antibodies was performed with 0.1% glutardialdehyde in TBS for 5 min and stopped by adding TBS containing 1% glycine and 1% lysine. Finally the beads were incubated over night at 4°C with the pooled lower sucrose gradient fractions (LF, 16–30). Beads containing the bound material were three times washed with TBS and incubated with ice-cold acidified acetone (acetone containing 125 mM HCl) for 30 min at 20°C. Elution was performed with different elution agents for 30 min. For Western blot analysis proteins were eluted with sample buffer containing 2% SDS. For MS analysis proteins were eluted with 100 mM triethylammonium bicarbonate (TEAB). The elution of PAZ proteins was supported by applying short ultrasonic pulses.

### Western blotting

For quantification of protein contents the BCA-assay kit (#23225; Pierce, Rockford, IL, USA) was applied. Immunopurified material was eluted from the beads with 2% SDS, 62.5 mM Tris, pH 6.8, prior to protein determination. The BCA kit tolerates up to 5% SDS, and 2% SDS are recommended to eliminate interference by lipids. Subsequently proteins were dissolved with sample buffer containing 2% SDS, 62.5 mM Tris, pH 6.8, 10% glycerol, and 0.01% bromophenol blue. Equal amounts of protein (100 ng) were resolved on a 15% Tris-glycine SDS-PAGE [20] and transferred onto nitrocellulose membrane (GE Healthcare) using semi-dry blotting techniques (BioRad). Membranes were blocked with 5% skimmed milk powder in PBS/T (123 mM NaCl, 7.4 mM Na_2_HPO_4_, 4.3 mM KH_2_PO_4_, 0.1% Tween20) for 1 h. Incubation with the respective primary antibody was performed over night at 4°C followed by a second blocking step with 5% skimmed milk powder (five times, 10 min each), subsequent incubation with the respective HRP-conjugated secondary antibody (GE Healthcare) and a final washing step in PBS/T (five times, 10 min each).

### Quantification and statistics

Immunoblots were incubated with Western Lightning ECL substrate and visualized using ImageQuant LAS 4000 (both GE Healthcare). Quantification of immunosignals was performed with samples obtained under identical experimental conditions (n = 3) and run in one gel. Pixel intensities of non-saturated bands (±SEM, standard error of the mean) from the same blot, were measured in voxels using ImageQuant TL software. Data were statistically processed employing unpaired Student’s t-test.

### Mass spectrometry—LC-MS/MS analysis of individual hippocampal PAZ

The immunopurified presynaptic active zone (PAZ) derived from mouse hippocampus was subjected to enzymatic digestion using the well-established serine protease trypsin. The amount of trypsin (Proteomics Grade, Sigma Aldrich, St. Louis, MO) was adjusted to an enzyme-to-substrate ratio of 1:50 for each sample according to the protein concentrations determined by BCA Protein Assay (Pierce, Thermo Scientific). The digestion was performed at 37°C for 18 h and stopped by adding 3 μL of formic acid (FA). Samples were dried down and solubilized in solvent A (5% MeCN, 0.1% FA) to obtain a final concentration of 1 μg peptide mixture per μL.

Peptide labeling with TMT sixplex was carried out according to the manufacturer’s protocol (TMT^6^, Thermo Scientific). The immunopurified hippocampal PAZ derived from the respective wild type mice was labeled with TMT-126, TMT-127 and TMT-128, whereas the immunopurified hippocampal PAZ from APP mutants was labeled with TMT-129, TMT-130, and TMT-131. The peptide mixtures were combined, purified and desalted by employing Pierce C18 spin columns (Thermo Scientific) and finally dried down. Prior to separation the sample was solubilized in solvent A (2% MeCN, 0.1% formic acid) to a final concentration of 1 μg/μl.

Chromatographic separation of the mixture was performed on a Dionex Ultimate 3000 RSLC nano system (Thermo Scientific). Peptides were trapped on an Acclaim PepMap 100 μCartridge Column C18, 300 μm x 0.5 cm, 5 μm, 100 Å (backflush mode) trapping column prior to separation on an Acclaim PepMap 100 C18 (2 μm, 100 Å, 75 μm i.d. x 50 cm) EASY-Spray nano column at a flow rate of 250 nL/min. A gradient of 150 minutes with increasing amounts of solvent B (80% MeCN, 0.08% FA) was applied. The LC system was coupled online to a Q-Exactive Plus hybrid quadrupole-Orbitrap instrument (Thermo Scientific) and operated in a data-dependent acquisition mode selecting the top 12 most intense peaks from a survey scan for fragmentation.

The survey scan was performed using the following parameters: scan range between 300–1500 m/z at a resolution of 70000 at m/z 200. The AGC target value was set to 3e6. MS/MS scans were acquired at a resolution of 17,500 at m/z 200 with an AGC target of 1e5. The precursors were isolated with an isolation width of 1.6 Da and the normalized collision energy (NCE) was 35. Dynamic exclusion was set to 20s, peptide recognition mode was enabled while singly and charged state above 8 and unassigned precursor ions were disabled. The immunopurified hippocampal PAZs were analyzed in triplicate.

Data processing, database searches for protein identification and relative quantification were performed using Proteome Discoverer (V1.4.0.288, Thermo Scientific). Spectra were deconvoluted by the integrated software module and a signal-to-noise filter of 1.5 was applied. The database search was performed on an in-house Mascot server. Precursor mass tolerance was set to 10 ppm and fragment mass tolerance to 0.02 Da. Oxidation of methionine and deamidation of asparagine and glutamine were allowed as variable modifications and TMT was set as fixed modification for lysine residues and peptide N-termini. Spectra were searched against a database of murine proteins (SwissProt, released on 2014-02-19) and a two-level decoy search was performed, with a target FDR value of ≤ 1%.

For quantification, the mass window for peak integration tolerance was set to 20 ppm and the most confident centroid peak detection was applied. Only unique peptides were considered and the results were normalized on protein median by the built-in software module.

Only proteins that were identified in each experiment and present in the hippocampal PAZ of all animals (n = 12) were considered for further analysis.

### Bioinformatics

#### Interaction data and databases

Binary protein-protein interaction (PPI) data were extracted from protein interaction databases (February 2015) (additional information about PPI networks is provided in S1 Text). To achieve the highest possible coverage of interaction data we used various databases. The following databases were accessed via the Proteomics Standards Initiative Common Query Interface (PSICQUIC) web service (Aranda et al., 2011): Databases of Interacting Proteins (DIP) [21], IntAct [22], and the Molecular INTeraction database (MINT) [23]. DIP consists of experimentally determined PPI using manual and automatic curation. The IntAct molecular interaction database is an open source database system and analysis tool for molecular interaction data derived from literature curation or direct user submissions. MINT stores binary interaction data and in addition considers indirect relationships and was recently merged into IntAct [24]. Data were returned and stored in the Human Proteome Organization Proteomics Standards Initiative—Molecular Interactions (HUPO PSI-MI) Tab 2.5 format. In this text format one binary interaction is stored per line. Supplementary data for each interaction is stored in 15 columns and contains, for example, information regarding both interactors, alternative identifiers or accession numbers for both identifiers, literature references, detection method, interaction type and cross references to other databases. The protein-interaction databases Biological General Repository for Interaction Datasets (BioGRID) [25], mentha: the interactome browser (mentha) [26], iRefIndex [27], and the Search Tool for the Retrieval of Interacting Genes/Proteins (STRING) [28] were downloaded manually. BioGRID is compiled from high throughput datasets and individual studies. The mentha database integrates experimentally determined direct PPI data from primary databases in compliance with the International Molecular Exchange Consortium (IMEx) curation policy [29] using the PSICQUIC protocol. iRefIndex consolidates PPI data from a variety of other protein interaction databases. The STRING database collects PPI data using computational methods like text mining and prediction algorithms. The data collection step created data files which contain also interactions between proteins of the dataset and proteins that are not members of the dataset. Only interactions where both interactors are members of the dataset were kept for further analysis. Respective UniProt accession numbers were used as unique identifiers for the proteins in the network. UniProt identifiers were mapped onto the BioGRID and STRING interactions using respective identifier mapping data from each database.

### Creating the network

Cytoscape 3.2.1 [30], an open source software platform for visualizing, integrating, and analyzing networks, was used to create the actual PPI network. All data files were imported into Cytoscape and the networks were merged into a single network using Cytoscape’s network merging tool. Filters were applied to retain only physical interactions which were experimentally detected. We used a selection of proteins that are constituents of the hippocampal PAZ. Singletons and small connected components which had no connection to the giant connected component were excluded. Additional node information was loaded from UniProt and the relative abundance was attributed to the respective node. Localization of proteins was derived from UniProt and mapped manually onto each node. The Cytoscape 3 session file of or data set is provided as S1 Cytoscape 3 File.

### Network analysis, community structure, and visualization

The Cytoscape NetworkAnalyzer tool was used to analyze the topology of the network and to compute centrality values for each node. The importance of nodes in the network was ranked in accordance with their centrality values. Centralities are numerical values assigned to nodes based on statistical properties. We used the clustering coefficient, degree, and shortest path betweenness as implemented in the Cytoscape NetworkAnalyzer tool. The cluster coefficient describes the densities of connections between neighbors with a range from 0 to 1 the latter with the highest density (100%), degree is the number of interactions, and betweenness represents the ratio of all shortest paths between two nodes to the number of shortest paths a given node (e.g. APP) is participating in. Community detection was performed for unweighted modularity [31,32] using Radatools 3.2 [33] that outputs the best partition found in form of a text file which contains information about the number of elements in each partition, a list of elements, the size of each community, and the indices of each element. The Radatools software implements several algorithms for the optimization of modularity. Here, a combination of tabu search [33], extremal optimization [34], fast heuristics [35] and spectral optimization [36] was used. To visualize the distributions of up- and downregulated proteins in APP-KO and NexCre-cDKO the relative abundances of all proteins were mapped on the network. We used Cytoscape 3.2.1 for all network layouts and visualizations.

All network visualizations are optimized for color vision deficiency. Changes in protein abundance of more than ±10% are reflected by increasing sizes of the nodes.

## Results

We have previously identified all APP family members as constituents of the presynaptic active zone (PAZ) and provided evidence for the physiological relevance of APP at neurotransmitter release sites [11,37]. In this study we analyzed the hippocampal PAZ proteome derived from APP single KO mice and conditional APP/APLP2 double knockout mice (NexCre-cDKO) as compared to the respective controls. To unravel APP as a structural and functional regulator of the hippocampal PAZ proteome we developed a PPI network.

**Fig 1:**
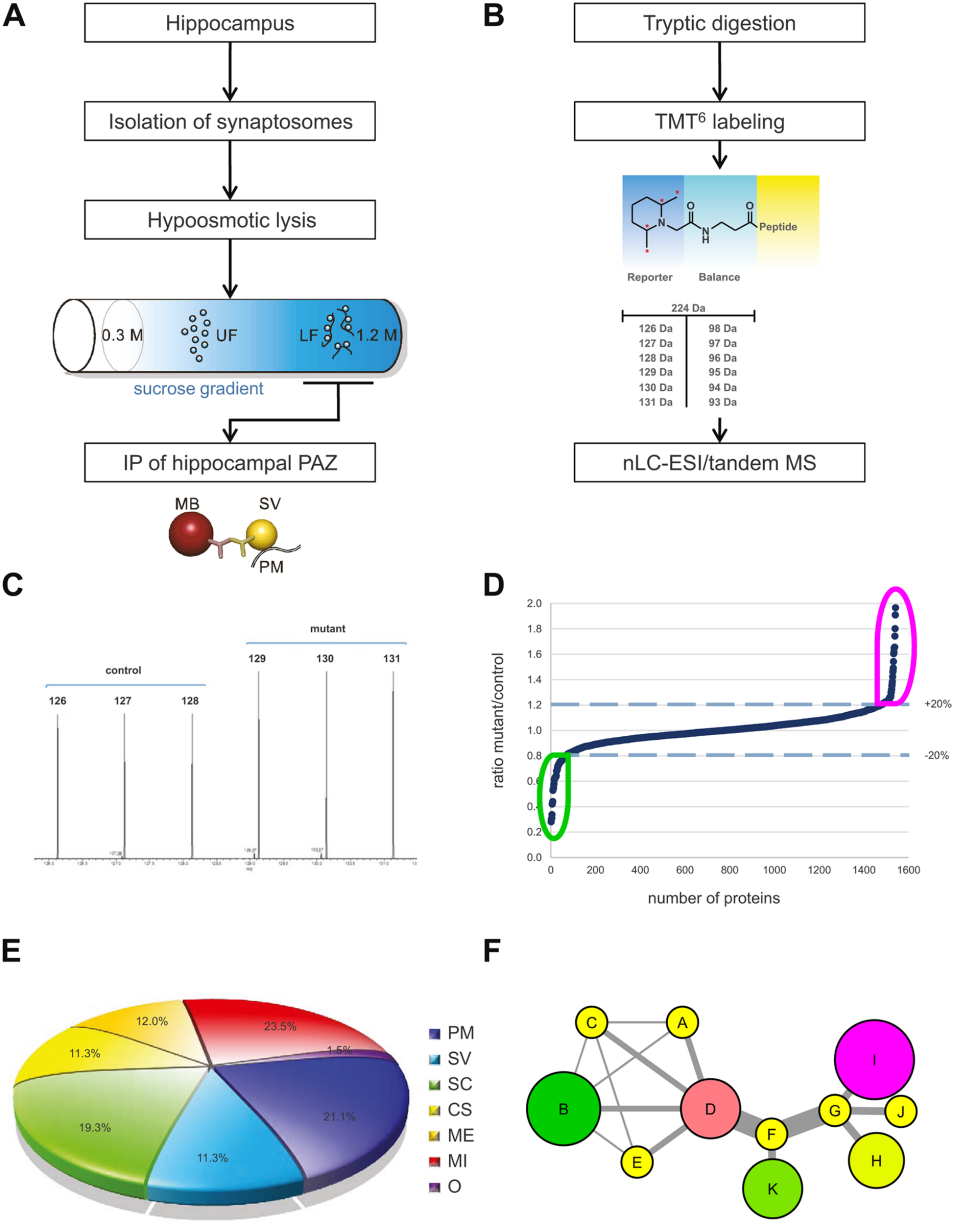
Overview of the experimental design. A Workflow of subcellular fractionation and immunopurification of the native hippocampal PAZ. B Experimental outline of isobaric labeling of peptides with TMT^6^ and MS analysis by nano-high-pressure liquid chromatography (nHPLC-ESI). C Example of peptide signals (m/z) for the six reporter groups. D Differences in protein abundance of hippocampal PAZ constituents between APP-mutant and control. E Pie chart diagram of proteins attributed to the PAZ. F Scheme of a PPI network illustrating proteins (exemplarily designated as A-K) as nodes and edge betweeness. The thickness of the connections represents the importance of the respective edges for information flow within the network (edge betweenness). Change in abundance of more than ±10% is reflected by increasing sizes of nodes. The color code corresponds to the degree of up- (magenta) and downregulation (green). Nodes in yellow represent proteins with changes in abundance of less than ±10%. UF, upper fractions; LF, lower fractions; IP, immunopurification, MB, magnetic bead; PM, plasma membrane; SV, synaptic vesicle, SC, signaling cascade; CS, cytoskeleton; ME, metabolic enzymes; MI, mitochondria; O, others.

The native hippocampal PAZ from age-matched individual mice was subjected to subcellular fractionation and immunopurification (Fig 1A) [14]. Importantly, the synaptic vesicle protein SV2, the target protein applied for immunopurification, revealed no change in abundance in either APP-mutant (S1 Table; APP-mutant/control 0.997). For the setup of the PPI-network, we analyzed the experimental data derived from APP single KO mice and conditional APP/APLP2 double knockout mice (NexCre-cDKO) as compared to the respective controls. To identify changes in protein abundance between two or more biological conditions, we employed an isobaric labeling approach (tandem mass tag, TMT^6^) combined with high-resolution mass spectrometry (Fig 1B). The abundance of reporter tags reflects the ratio of peptides in biological triplicates and was used for quantification (Fig 1C). Significantly identified proteins were plotted in an ascending order according to their changes in protein abundance (mutant/control) (Fig 1D). A common core proteome (~1100 proteins) was defined, including only those proteins being identified in all animals (n = 12) and in all experiments (Fig 1E and S1 Table). Furthermore, these core proteins were categorized into different groups within the hippocampal PAZ and were assigned as follows: integral and associated presynaptic plasma membrane proteins (PM), integral and associated synaptic vesicle proteins (SV), signaling cascade (SC), cytoskeleton (CS), metabolic enzymes (ME), mitochondria (M) and yet uncharacterized proteins named others (O) (Fig 1E). The percentage composition of the different categories is depicted in the pie chart (Fig 1E). All proteins within the pie chart were further filtered and visualized according to their subcellular localization and their functional allocation (clustering) by creating a PPI network (scheme Fig 1F; additional information about PPI networks is provided in S1 Text), excluding all non-physical and non-validated interactions.

**Fig 2:**
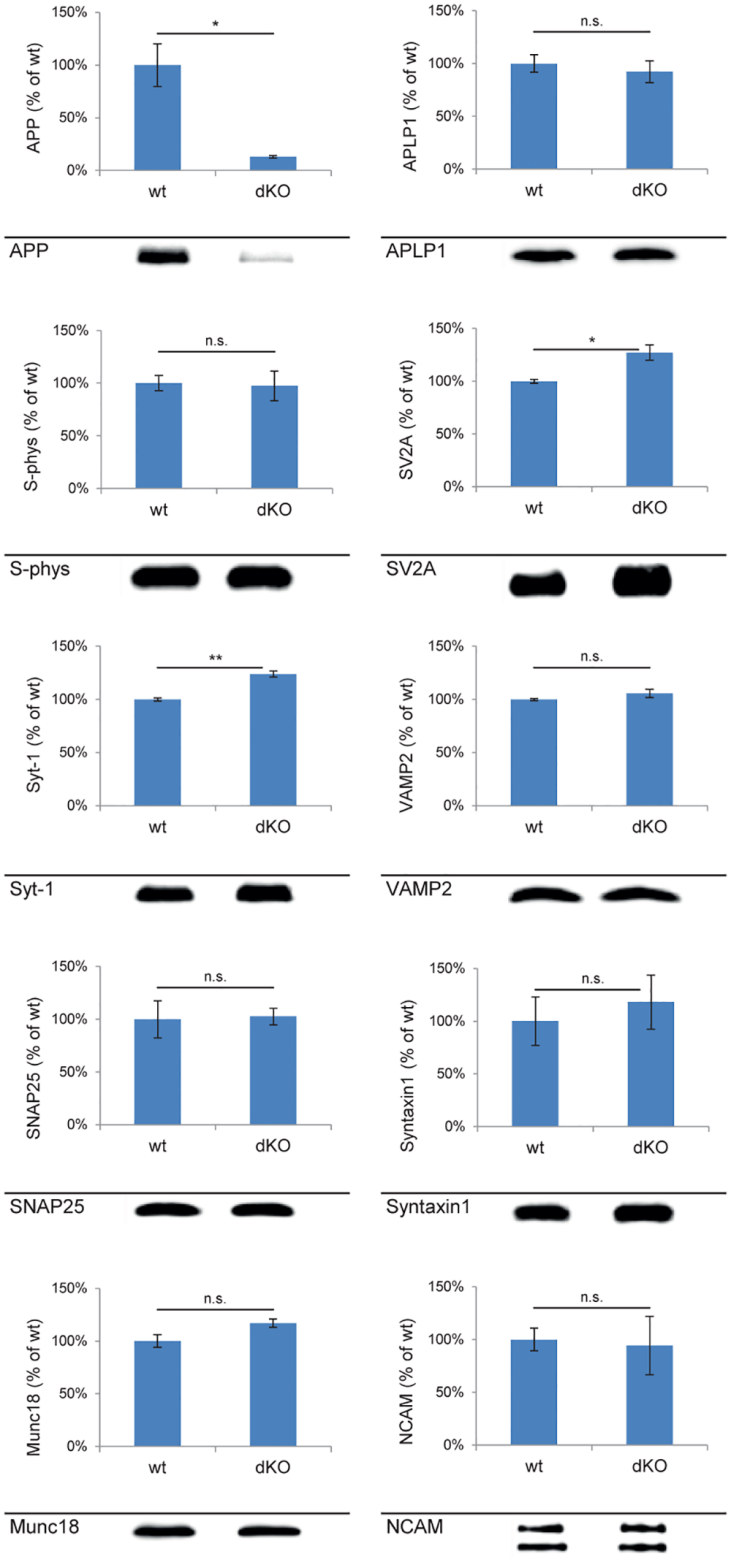
Quantitative immunodetection of hippocampal PAZ constituents derived from wild type and NexCre-cDKO mice. Bar graphs are mean ± SEM, n = 3. *p<0.05, **p<0.01, n.s. difference not significant. The control (APLP2-KO, wt) was set to 100%. A representative Western blot is shown below each diagram. Equal amounts of protein (150 ng) were loaded per lane. APP (100±20%, 13±1%; 110 kDa), APLP1 (100±8%, 92±10%, 90 kDa), Munc18 (100±6%, 117±4%, 68 kDa), NCAM (100±11%, 94±28%, 140 and 180 kDa), SNAP25 (100±17%, 103±8%, 25 kDa), SV2A (100±2%, 127±7%, 86 kDa), synaptotagmin-1 (100±1%, 124±3%, Syt-1, 65 kDa), synaptophysin (100±7%, 97±14%, S-phys, 38 kDa), syntaxin1 (100±23%, 118±26%, 34 kDa), VAMP2 (100±1%, 106±4%, 18 kDa). Note that in NexCre-cDKO the deletion of APP is restricted to excitatory neurons.

Recently, we analyzed the impact of single KO of either APP, ALPL1 or APLP2 on selected PAZ proteins derived from individual mouse brains [37] and developed a novel experimental setup to isolate the native PAZ from distinct brain regions such as olfactory bulb, cerebellum and hippocampus [14]. Employing quantitative immunodetection we now analyzed the NexCre-cDKO for selected members of the hippocampal PAZ proteome including APP, APLP1, the synaptic vesicle proteins synaptophysin (S-phys), SV2A, synaptotagmin-1 (Syt-1) and the SNARE complex members vesicle-associated membrane protein 2 (VAMP2), synaptosomal-associated protein 25 (SNAP25), syntaxin-1 (Syt-1) whereby the single APLP2-KO served as control (Fig 2). In agreement with our previous analysis [7] APP protein expression is reduced to about 10% of control in hippocampus whereas APLP1 remains unaltered. Similarly, the abundance of synaptophysin, VAMP2, SNAP25, syntaxin1, Munc18, and the neural cell adhesion molecule NCAM is unchanged in the hippocampal PAZ as compared to control. SV2A and synaptotagmin-1 are slightly increased in mutant mice.

### Network analysis

**Fig 3:**
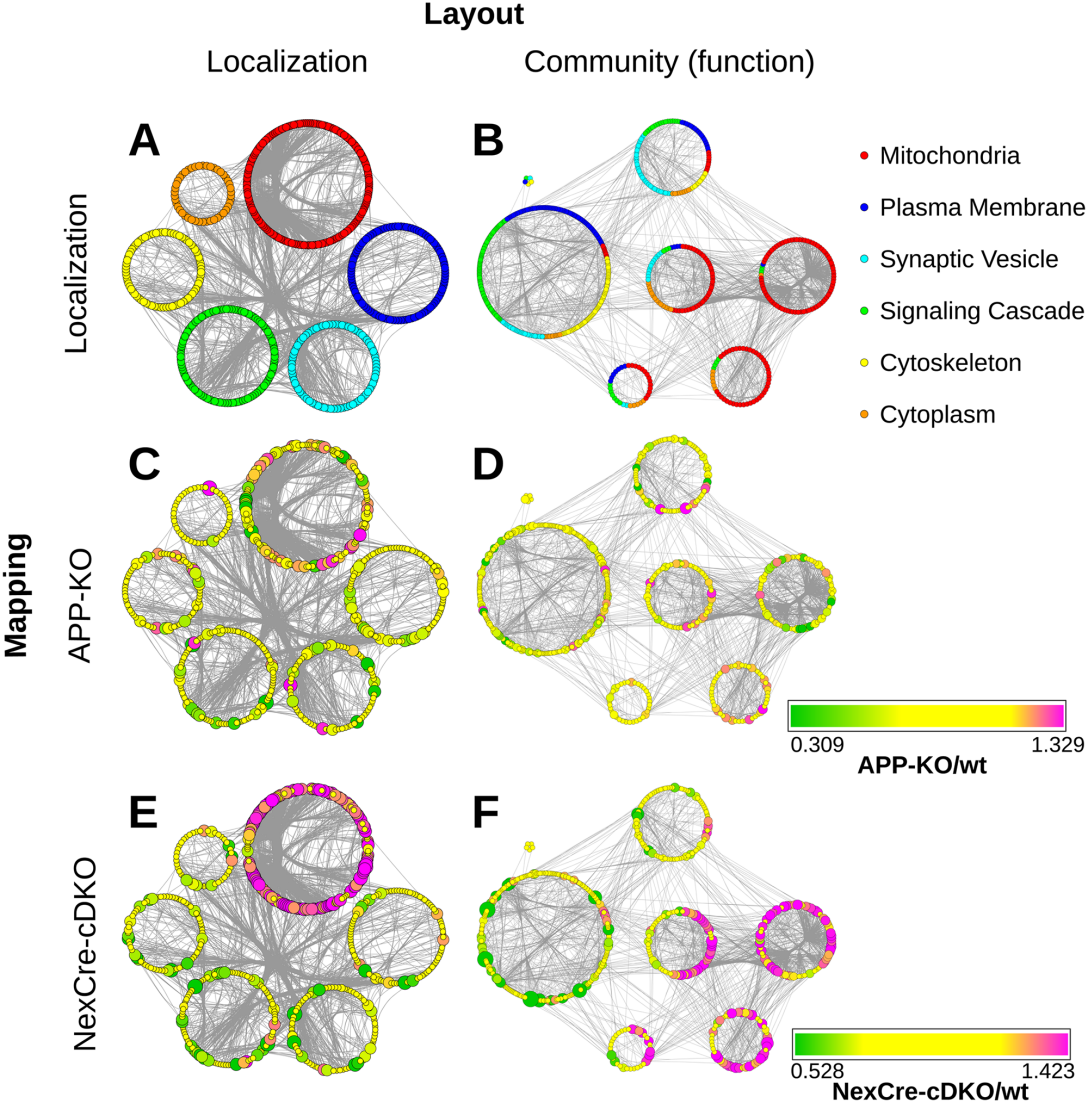
The interactome of the native hippocampal PAZ core proteome. A Proteins grouped according to their localization (localization layout). B Community structure layout (function) of the network. The size of the rings corresponds to the respective number of proteins. The color code corresponds to the pie chart diagram (cf. Fig 1E). C Impact of APP deletion on relative protein abundance mapped according to their localizations. D Impact of the APP-KO on relative protein abundance mapped according to the community structure. E Impact of the NexCre-cDKO on relative protein abundance mapped according to their localizations. F Impact of the NexCre-cDKO on relative protein abundance mapped according to the community structure. Change in abundance of more than ±10% is reflected by increasing sizes of nodes. The color code corresponds to the degree of up- (magenta) and downregulation (green). Nodes in yellow represent proteins with changes in abundance of less than ±10%. Each node (dot in the rings) within this network represents a protein and each edge (connection) represents a reported physical interaction between two proteins. Edges are bundled for clarity.

**Fig 4:**
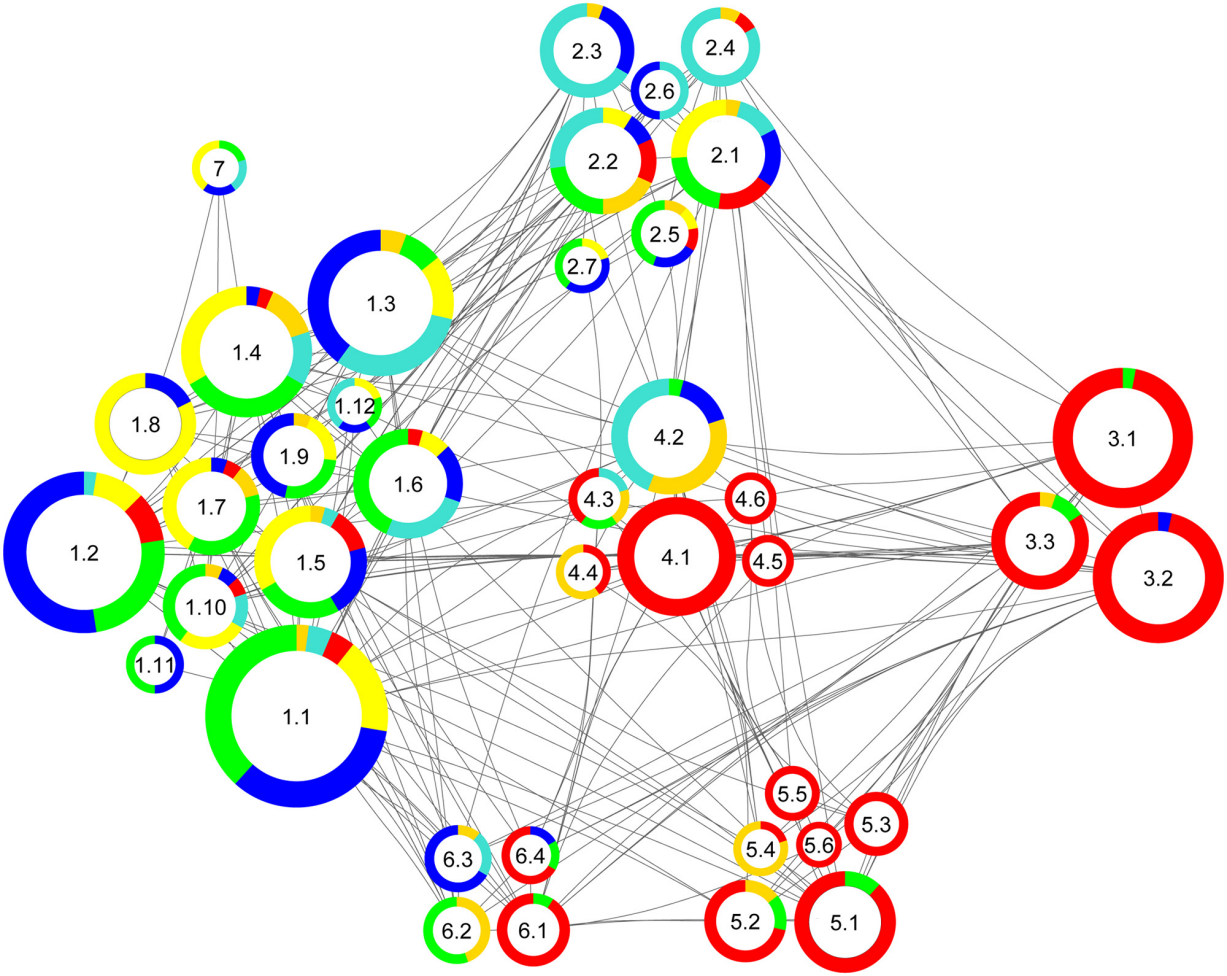
Subcommunity structure of the network based on Fig 3B. The color code corresponds to the pie chart diagram (cf. Fig 1E). The size of the rings corresponds to the respective number of proteins. Communities (1–7) were subdivided into the following functional clusters (e.g. 1.1–1.12): 1.1 Calcium Signaling, 1.2 Presynaptic Membrane, 1.3 Exocytosis, 1.4 Cytoskeleton Organization, 1.5 Membrane Assembly, 1.6 G-Protein Signaling, 1.7 Organelle Transport, 1.8 Actin Organization, 1.9 Synapse Assembly, 1.10 Membrane Regulation, 1.11 Inhibitory Regulation, 1.12 Vesicle Priming, 2.1 Membrane Trafficking, 2.2 Vesicle Budding, 2.3 Endocytosis, 2.4 Proton Transport, 2.5 Phospholipid Metabolism, 2.6 Vesicle Organization, 2.7 Membrane Organization, 3.1 Electron Transport Chain, 3.2 Energy Metabolism, 3.3 Stress Defense, 4.1 Cellular Respiration, 4.2 Glycolysis, 4.3 Guanine Metabolism, 4.4 Glycerol Metabolism, 4.5 Mitochondrial Metabolism, 4.6 Mitochondrial Assembly, 5.1 Fatty Acid Catabolism, 5.2 Neurotransmitter Metabolism, 5.3 Amino Acid Catabolism, 5.4 Metabolism, 5.5 Fatty Acid Metabolism, 5.6 Mitochondrial Targeting, 6.1 Mitochondrial Protein Trafficking, 6.2 Heatshock Response, 6.3 Neuronal Regulation, 6.4 Protein Folding, 7 Functional Dynamics.

**Fig 5:**
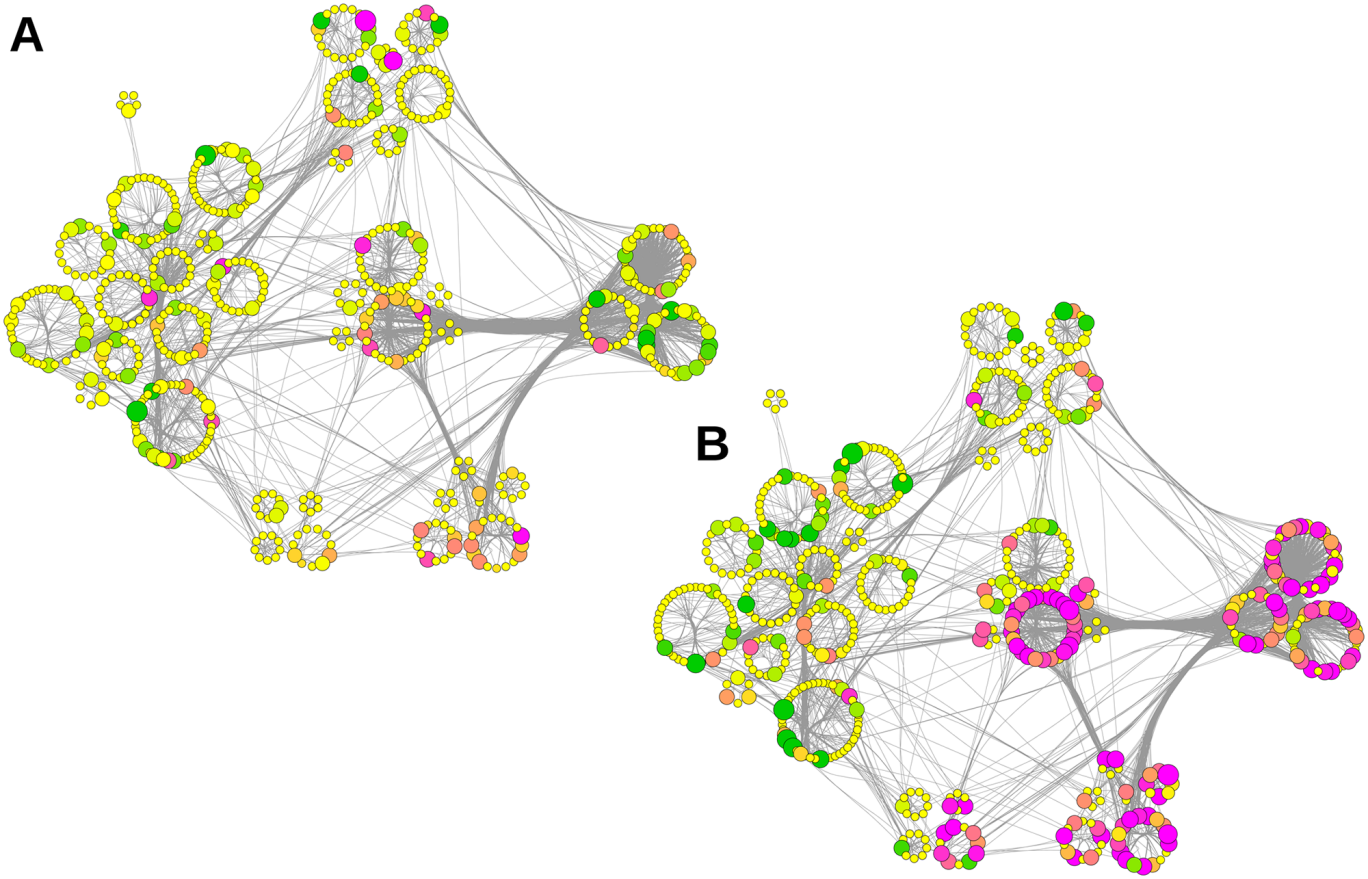
Relative abundance of proteins mapped to the subcommunity structure of the network. The size of the rings corresponds to the respective number of proteins. Communities were subdivided into functional clusters according to Fig 5. A Impact of APP deletion. B Impact of the NexCre-cDKO. Change in abundance of more than ±10% is reflected by increasing sizes of nodes. The color code corresponds to the degree of up- (magenta) and downregulation (green). Nodes in yellow represent proteins with changes in abundance of less than ±10%.

**Fig 6:**
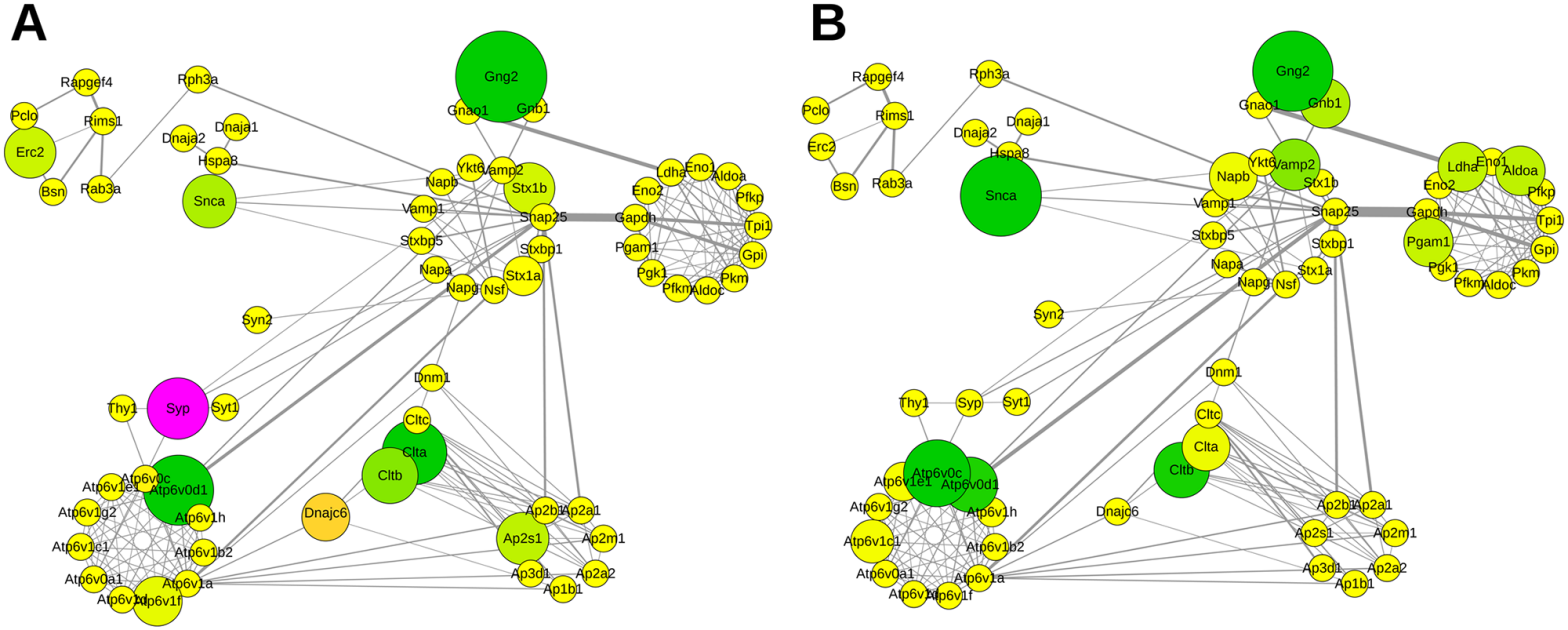
Relative abundance of proteins mapped to the functional subnetwork of the synaptic vesicle cycle. A Impact of APP deletion. B Impact of the NexCre-cDKO. Change in abundance of more than ±10% is reflected by increasing sizes of nodes. The color code corresponds to the degree of up- (magenta) and downregulation (green). Nodes in yellow represent proteins with changes in abundance of less than ±10%. Abbreviations are the respective gene names of individual proteins as given in UniProt database and in the supplementary information S1 Table.

**Fig 7:**
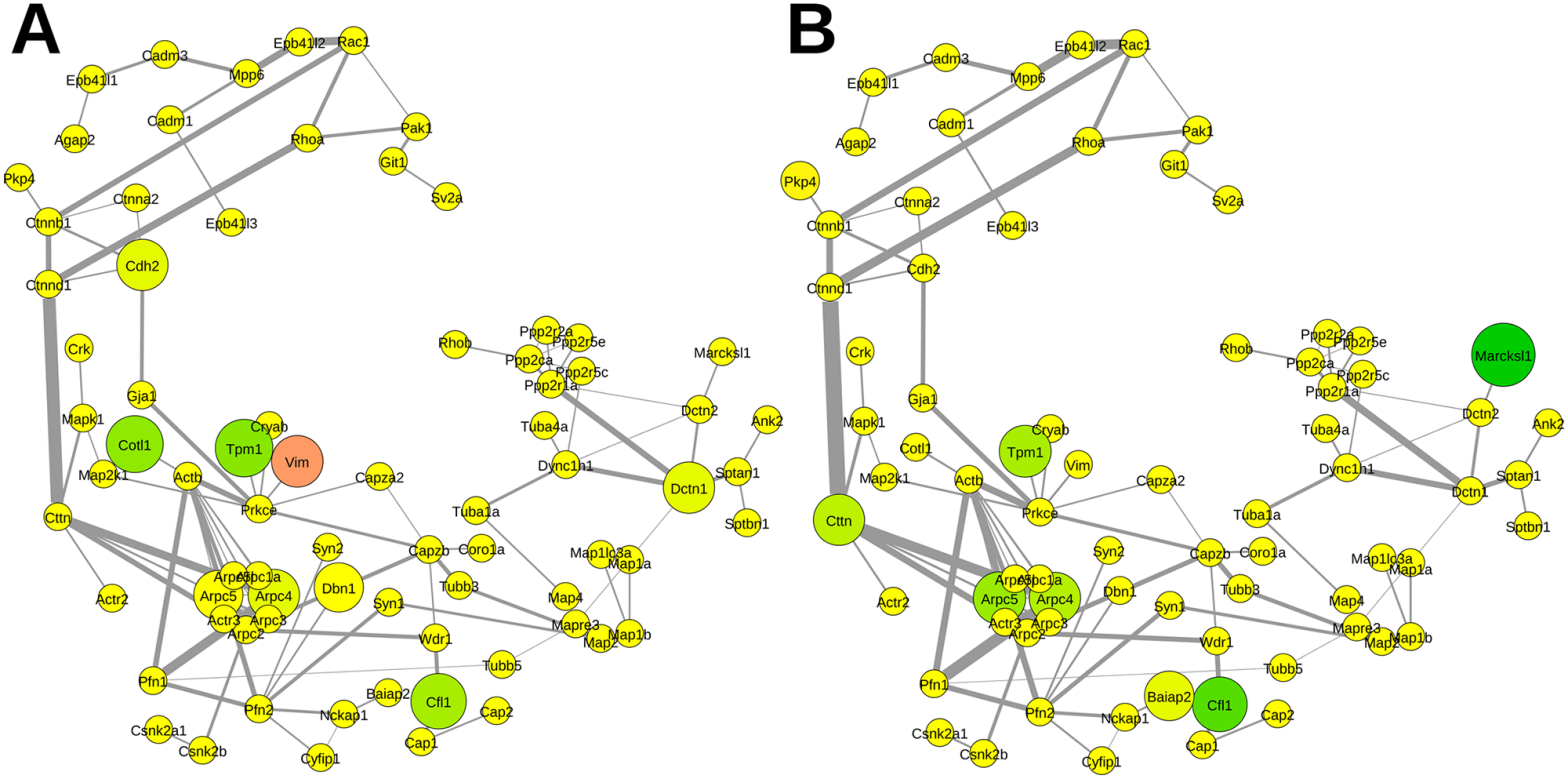
Relative abundance of proteins mapped to the functional subnetwork of the cytoskeleton organization. A Impact of APP deletion. B Impact of the NexCre-cDKO. Change in abundance of more than ±10% is reflected by increasing sizes of nodes. The color code corresponds to the degree of up- (magenta) and downregulation (green). Nodes in yellow represent proteins with changes in abundance of less than ±10%. Abbreviations are the respective gene names of individual proteins as given in UniProt database and in the supplementary information S1 Table.

**Fig 8:**
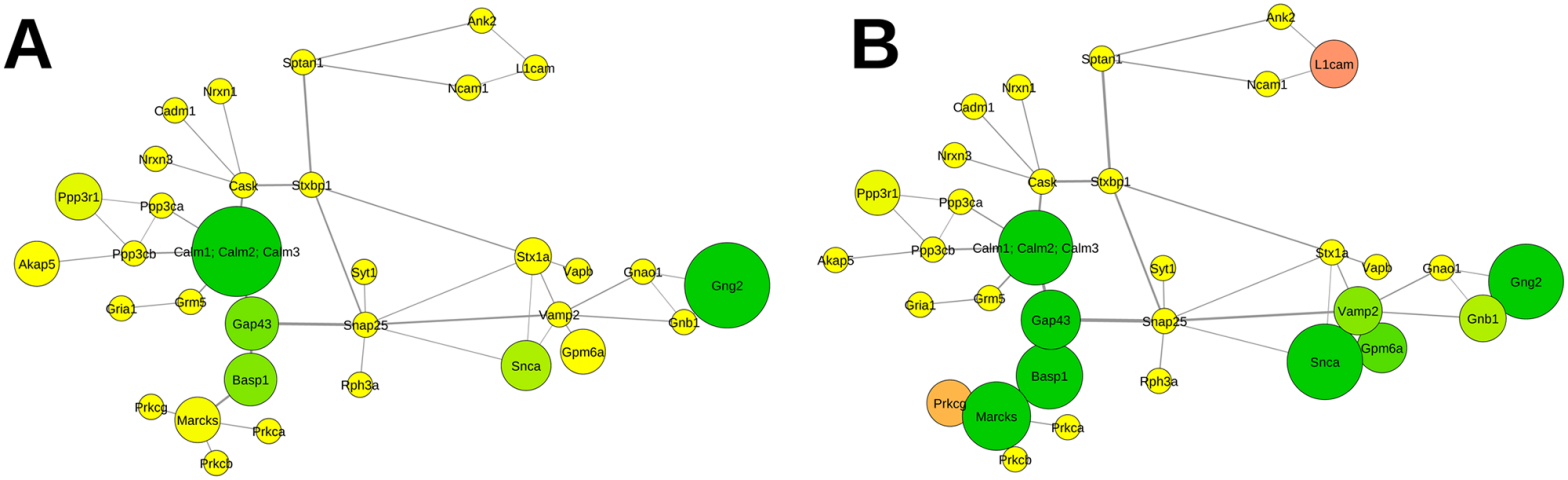
Relative abundance of proteins mapped to the functional subnetwork of calcium homeostasis. A Impact of APP deletion. B Impact of the NexCre-cDKO. Change in abundance of more than ±10% is reflected by increasing sizes of nodes. The color code corresponds to the degree of up- (magenta) and downregulation (green). Nodes in yellow represent proteins with changes in abundance of less than ±10%. Abbreviations are the respective gene names of individual proteins as given in UniProt database and in the supplementary information S1 Table.

To further characterize the impact of APP deletion the abundance of hippocampal constituents was visualized according to both their subcellular localization (Fig 3A, left) and their functional allocation (Fig 3B, right). The final network layout illustrates proteins as nodes and interactions (experimentally validated physical interactions) between proteins as edges (Figs 3–8). The resulting 615 proteins revealed a high degree of networking, confirming their direct association with the hippocampal PAZ core proteome.

The separation into localization and function allowed us to gain further insights into the topological connectivity of single proteins and the organization of individual proteins within the proteomic network (Fig 3). Localization, embodied by six groups according to the pie chart layout, highlights all structural core constituents of the PAZ network (color, protein number): mitochondria (red, 188), plasma membrane (blue, 108), synaptic vesicle (turquoise, 81), signaling cascade (green, 113), cytoskeleton (yellow, 79), and metabolic enzymes (orange, 46) (Fig 3A). The term function in a network is associated with topological features that allow insight into the connectivity and relationship not only of single proteins but also insight into the functional organization of a proteome (community structures). A community is a densely connected subnetwork within the PPI network of the PAZ and represents a functional module within the entire community structure. To find a linkage between proteins and their functions within the network we applied a community detection algorithm resulting in seven communities (Fig 3B).

In the next step, we mapped the relative abundance of each protein within each layout to visualize the impact of APP deletion on the PPI-network (Fig 3C–3F, APP-KO Fig 3C and 3D, NexCre-cDKO Fig 3E and 3F). Proteins affected by the deletion were highlighted in magenta (upregulation) or green (downregulation), whereas slightly affected proteins display a color-gradient and non-affected proteins are in yellow (Fig 3C–3F). Within the localization and community structure network, APP deletion displays a wide distribution of up- and downregulated proteins (Fig 3C and 3D) whereas deletion of APP in NexCre-cDKO revealed a defined pattern allocating significantly dysregulated proteins to individual groups (e.g. mitochondria, Fig 3E and 3F).

The community structure provided by Radatools does not resolve the functional structure unambiguously. Within the community structures individual protein groups (functional clusters) are represented, e. g. synaptic vesicle exo- and endocytosis, Ca^2+^-homeostasis and cytoskeleton. Therefore, each community structure consists of a collection of proteins that belongs to individual functional cluster. This collection of proteins can be summarized under the term heterogeneity to emphasize this complex and diverse layout of the community structures. For better visualization of this “heterogenic nature”, we created a subcommunity layout representing all individual functional clusters (Fig 4).

Proteins of the respective functional cluster are listed in S1 Table (Worksheet SubCom—Subnet)

In addition, we mapped the relative abundance of each protein within the subcommunity structure layout to visualize the impact of APP deletion on the PPI network (Fig 5A, APP-KO left, Fig 5B, NexCre-cDKO right). The visualization of functional units within this network allows distinguishing differently affected clusters such as vesicle organization (2.6 in Fig 4) and energy metabolism (3.2 in Fig 4). Several members involved in energy metabolism are downregulated at the hippocampal PAZ in APP-KO mice; in contrast the majority is upregulated in NexCre-cDKO.

The analysis of subcommunity structure was a prerequisite to define the impact of APP deletion on distinct functions including synaptic vesicle cycle, cytoskeletal organization and calcium homeostasis within the hippocampal PAZ proteome. For this purpose subnetworks were created containing APP and its family members APLP1 and APLP2 (S1–S3 Figs). Remarkably APP appears as a highly connected node with 70 interactions within this network with a clustering coefficient of 0.0402 and a betweenness of 0.1198 indicating a central regulating function. In the synaptic vesicle cycle APP represents a hub that interacts with 22 proteins, in cytoskeleton organization with 18 proteins, and in calcium homeostasis with 20 proteins. The cluster coefficient that describes the densities of connections between neighbors in the synaptic vesicle cycle is 0.1125 with a betweenness of 0.4172 indicating a regulating role of APP within the network. Values for cytoskeleton organization were 0.0909 with a betweenness 0.2529 and for calcium homeostasis 0.0684 with a betweenness 0.7049 indicating that APP is not part of the cluster but a linker monitoring information flow.

Subsequently, the impact of APP deletion was mapped onto these subnetworks (APP-KO left, NexCre-cDKO right). The thickness of the connections represents the importance of the respective edges for information flow within the network (edge betweenness).

### Subcommunity structure—synaptic vesicle cycle

In the synaptic vesicle cycle (Fig 6A)–comprising members of the cytomatrix of the active zone, SNARE complex, G-proteins, glycolysis, endocytosis, and vATPase—several proteins were downregulated (given as ratio APP-KO/control±variability in percent). These include α-synuclein (83.6±4.2%, Snca), syntaxin-1B (86.5±0.7%, Stx1b), ERC protein 2 (85.9±2.4%, Erc2), guanine nucleotide-binding protein G(I)/G(S)/G(O) subunit gamma-2 (37.0±5.8%, Gng2), clathrin light chain A (69.9±11.4%, Clta) and B (80.4±2.3%, Cltb), AP-2 complex subunit sigma (84.9±19.1%, Ap2s1), and v-type proton ATPase subunit d 1 (62.9±6.4%, Atp6v0d1) and only a few were upregulated (given as ratio APP-KO/control±variability in percent) such as putative tyrosine-protein phosphatase auxilin (111.7±4.2%, Dnajc6) and synaptophysin (124.7±2.5%, Syp) while the majority remained unaltered in APP-KO mice (Fig 6A).

In contrast, hippocampal PAZ proteins derived from NexCre-cDKO were mainly downregulated (given as ratio NexCre-cDKO/control±variability in percent) (Fig 6B). The mediator of SNARE complex formation α-synuclein (52.8±3.7%, Snca) is severely downregulated whereas members of the SNARE complex the vesicle-associated membrane protein 2 VAMP2 (85.3±2.1%, Vamp2), the synaptosomal-associated protein 25 SNAP25 (99.7±0.8%, Snap25), syntaxin-1A (104.3±1.3%, Stx1a), syntaxin-1B (98.7±1.2%, Stx1b) are less or not affected. In addition, downregulated proteins comprise guanine nucleotide-binding protein G(I)/G(S)/G(O) subunit gamma-2 (53.6±11.0%, Gng2), guanine nucleotide-binding protein G(I)/G(S)/G(T) subunit beta-1 (87.0±5.5%, Gnb1), members of the glycolysis cycle the L-lactate dehydrogenase A chain (87.6±2.2%, Ldha), fructose-bisphosphate aldolase A (87.5±1.6%, Aldoa), phosphoglycerate mutase 1 (87.8±2.5%, Pgam1), clathrin light chain A (89.3±3.6%, Clta) and B (81.0±1.4%, Cltb), the v-type proton ATPase 16 kDa proteolipid subunit (68.3±3.1%, Atp6v0c), and v-type proton ATPase subunit d 1 (81.3±2.5%, Atp5v0d1) (Fig 6B).

### Subcommunity structure—cytoskeletal organization

APP is strongly interconnected with proteins governing cytoskeletal organization (S2 Fig). Important connections for the information flow within this network are via actin and the microtubule motor dynein. Several organizers of the cytoskeleton are affected by APP deletion (Fig 7A) such as coactosin-like protein (86.3±4.4%, Cotl1), cofilin-1 (83.1±4.7%, Cfl1), tropomyosin alpha-1 chain (80.6±2.5%, Tpm1), and vimentin (113.9±2.6%, Vim). Especially in the NexCre-cDKO (Fig 7B) the MARCKS-related protein (74.3±7.1%, Marcksl1) that interacts with calmodulin, and actin is downregulated. Other affected proteins are cofilin-1 (83.5±2.2%, Cfl1), tropomyosin alpha-1 chain (86.7±2.8%, Tpm1), actin-related protein 2/3 complex subunit 4 (87.1±0.4%, Arpc4) and subunit 5 (86.1±1.0%, Arpc5), and the src substrate cortactin (87.4±4.4%, Cttn).

### Subcommunity structure—calcium homeostasis

The protein network of calcium homeostasis reveals that APP is a central regulator (S3 Fig) that is interconnected with e.g. bassoon, neural cell adhesion molecule 1, neural cell adhesion molecule L1, neuromodulin, SNAP-25, and neuroplastin. In the hippocampal PAZ derived from APP-KO mice neuromodulin (79.0±1.0%, Gap43), the guanine nucleotide-binding protein G(I)/G(S)/G(O) subunit gamma-2 (37.0±5.8%, Gng2), α-synuclein (83.6±4.2%, Snca), brain acid soluble protein 1 (80.1±4.3%, Basp1), and calmodulin (30.9±17.8%, Calm) are downregulated (Fig 8A). Mapping the change in protein abundance derived from NexCre-cDKO demonstrates the downregulation of calmodulin (54.2±14.3%, Calm) with its modulator neuromodulin (72.0±2.6%, Gap43), brain acid soluble protein 1 (63.6±1.1%, Basp1), myristoylated alanine-rich C-kinase substrate MARCKS (61.3±8.0%, Marcks), α-synuclein (52.8±3.7%, Snca), vesicle-associated membrane protein 2 VAMP2 (85.3±2.1%, Vamp2), neuronal membrane glycoprotein M6-a (83.4±8.5%, Gpm6a), guanine nucleotide-binding protein G(I)/G(S)/G(T) subunit beta-1 (87.0±5.5%, Gnb1), and the guanine nucleotide-binding protein G(I)/G(S)/G(O) subunit gamma-2 (53.6±11.0%, Gng2). Neural cell adhesion molecule L1 (114.0±3.1%, L1cam) and protein kinase C gamma type (112.6±1.1%, Prkcg) reveal an increase in protein abundance (Fig 8B).

## Discussion

In this study, we combined state-of-the-art proteomics and bioinformatics to unravel the impact of APP deletion on hippocampal neurotransmitter release sites. We present a core-proteome of the hippocampal PAZ that consists of proteins that can be identified in all APP-mutants. This allowed us to trace alterations within the proteomic composition resulting from the deletion of APP. Proteins were structurally and functionally sorted and related to their physiological tasks. The analysis of APP mutant mice revealed that deletion of APP results in changes in abundance of proteins involved in synaptic vesicle exocytosis, cytoskeletal organization and calcium-signaling. In this context, we address the question, how APP is functionally integrated into these subcommunities of proteins.

### APP is a hub at the active zone

**Fig 9:**
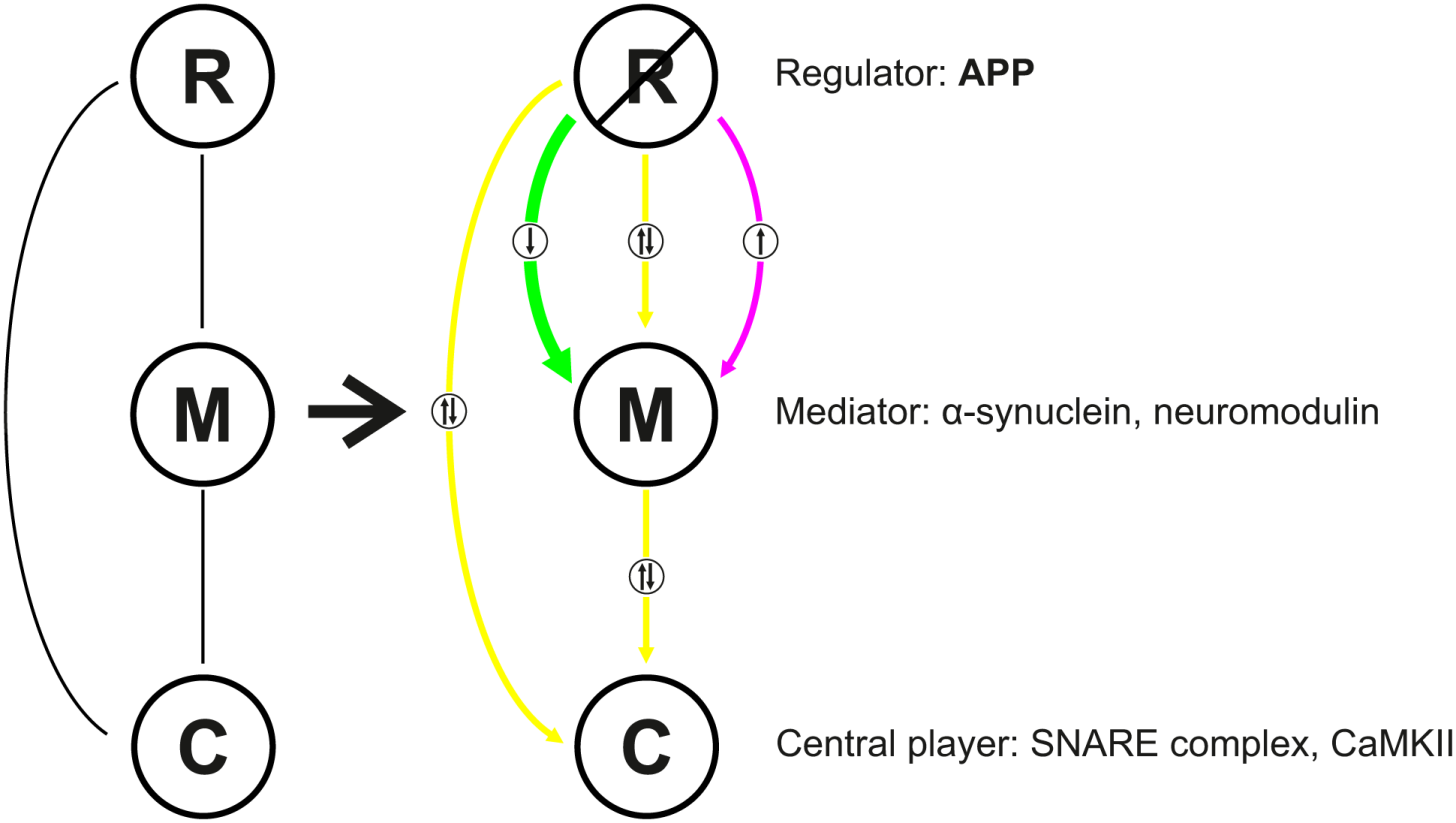
Scheme illustrating the regulatory role of APP in a context-sensitive manner at the hippocampal PAZ. R, regulator; M, mediator, C, central player. Color code: magenta, upregulation (in APP-mutants); green, downregulation (in APP-mutants); yellow, unaltered.

The application of network analysis by e.g. centrality and path length, visualization techniques, and functional analysis by subcommunity structures allowed insights into the changes of protein abundance following APP deletion. APP is highly interconnected in all networks analyzed and serves as a hub, implicating that it is important for the structure and function of the entire network. The high value for betweenness centrality indicates a bridging role of APP between the functional modules. The low values for the clustering coefficient supports the linking function of APP in the interaction network of the hippocampal PAZ. APP and its interactors are central in the information flow as indicated by the high value for edge betweenness. For example, APP interacts with bassoon physically [38] and is thereby embedded into the cytomatrix of the active zone (CAZ). Interactors of bassoon are in turn the ERC protein 2, piccolo and regulating synaptic membrane exocytosis protein 1. Deletion of APP leads to a decomposition and rearrangement of the entire network structure. This network is based on current database knowledge about physical interactions between proteins. Therefore APP deletion results in loss of network components. Interestingly, this is not reflected by a loss of biological functions. For example following APP deletion bassoon and its interactors are still part of the synaptic vesicle cycle. However they are no longer connected in cytoskeleton organization and calcium homeostasis subnetworks. In addition reorganization of the network results in changes in path lengths. For example in the synaptic vesicle cycle the vesicular proton pump vATPase and bassoon are linked via APP. Deletion of APP results in an increase in the shortest path length by three steps (interactions). Similarly, in calcium homeostasis loss of APP extends the shortest path length from extracellular cell adhesion (e.g. NCAM1) to trimeric G-proteins involved in intracellular signaling by three interactions. In contrast deletion of APP adds three additional interactions (4→7) from the homophilic cell-cell adhesion molecule 1, that acts in a calcium independent manner, to the actin-related protein complex. Based on our data, we hypothesize that APP functions as a regulator involved in all functional activities at the release sites. Depending on its respective microenvironment it may exert varying functions. This is illustrated in a scheme (Fig 9) which depicts the physical interaction between APP (regulator, R), mediators (M) and central players (C). Deletion of APP results in significant changes in the abundance of mediators but not of central players. For example the mediator of synaptic vesicle exocytosis α-synuclein [39] is downregulated in the mutants, however the central players the SNARE proteins VAMP2, SNAP25, and syntaxin1 [13] are not affected. In this context it is noteworthy to mention that deletion of SV2A does not affect the abundance of SNARE proteins but results in reduced SNARE complex formation [40]. Similarly, the mediators in calcium homeostasis calmodulin and neuromodulin are downregulated whereas the central player in learning and memory CaMKII as well as calcium-channels remain unaltered.

### APP regulates active zone players

Our data support the role of APP as a context-sensitive regulator of the hippocampal PAZ. This is in line with the current knowledge of the physiological function of APP in the CNS. Recently, APP has been suggested as a G-protein coupled receptor (GPCR) mediating presynaptic signaling and neurotransmitter release by activation of calcium-channels [41]. A selective interaction of APP with the neural cell adhesion molecule NCAM has been reported [42]. NCAM-induced activation of calcium-channels is a downstream effect of signaling through heterotrimeric G-proteins [43]. Our PPI network displays APP interaction via Munc18 with calcium/calmodulin activated Ser-Thr kinase CASK that is recruited to the cytosolic tail of neurexin-1 [44]. CASK may recruit calcium-channels to the presynapse [45] providing strong support that the presynaptic function of APP is mediated through similar mechanisms [46]. The APP-Mint1-Cask complex has also been implicated in neurexin-mediated signaling in presynaptic organization [47,48].

This complex is thought to play a regulatory role in synaptic vesicle release, and may be involved in development and maintenance of synaptic architecture [49]. The physical interaction of APP with bassoon [38] regulates the recruitment of ERC protein 2 (ELKS), RIM, and Munc13 that are involved in the recruitment of Ca^2+^-channels [13]. The regulatory impact of APP on the abundance of Ca^2+^-channels at the presynaptic plasma membrane [50] points to a functional integration of APP within this conserved network of the active zone key players. Furthermore, our data suggest, that APP is physically implemented into synaptic vesicles exocytosis. The link is provided by α-synuclein an essential mediator of SNARE-complex formation [39]. How APP and α-synuclein are connected to each other is still elusive and needs to be further investigated. However, embedding APP as a context-sensitive regulator into the active zone network supports the notion of a physiological role in synaptic transmission and plasticity.

Taken together, our findings provide a new perspective of the functional integration of APP into the hippocampal PAZ proteome. Furthermore, our network analysis incorporates APP into the evolutionary conserved active zone protein complex, comprising ELKS, CASK, as well as bassoon, Rim and Munc18. The coordinated arrangement of these proteins mediating synaptic vesicle docking and priming is essential for neuronal signaling. Interestingly, this subnetwork persists after APP deletion maintaining functional neurotransmission. We could demonstrate that deletion of APP differently affects individual PAZ subcommunities. Our results suggest, APP acts as a context-sensitive regulator within the presynaptic proteome linked to neuronal communication. Future studies have to reveal how APP deletion affects these subcommunities in a spatiotemporal way that eventually lead to impairments in learning and memory. These alterations may provide a molecular link to the pathogenesis of Alzheimer’s disease and open new strategies for therapeutic approaches.

## Supporting Information

S1 Table Hippocampal PAZ proteome. List of the hippocampal PAZ core proteome derived from APP-mutants, n = 12. Worksheet 1 “Overlap (APP)”: core constituents are sorted by the ratio APP-KO/wt in an ascending order. Worksheet 2 “Overlap (NexCre)”: core constituents are sorted by the ratio NexCre/wt in an ascending order). The graph displays the distribution of identified proteins according to their changes in abundance (APP/wt, and NexCre/wt).
Proteins of the respective functional cluster (Fig 4; *subcommunities structure*) are listed and their assignment to either *functional subnetwork* of the synaptic vesicle cycle (Fig 6), *functional subnetwork* of the cytoskeletal organization (Fig 7), or *functional subnetwork* of the calcium-homeostasis (Fig 8) is highlighted by an asterisk.
(XLSX)

S1 Fig Functional subnetwork of the synaptic vesicle cycle. *Functional subnetwork* of the synaptic vesicle cycle including APP and its family members APLP1 and APLP2. The color code corresponds to the pie chart diagram. Of note, APP appears as a highly connected node within this network. Abbreviations are the respective gene names of individual proteins as given in UniProt database and in the supplementary information S1 Table.
(PDF)

S2 Fig Functional subnetwork of the cytoskeleton. *Functional subnetwork* of cytoskeleton organization including APP and its family members APLP1 and APLP2. The color code corresponds to the pie chart diagram. Of note, APP appears as a highly connected node within this network. Abbreviations are the respective gene names of individual proteins as given in UniProt database and in the supplementary information S1 Table.
(PDF)

S3 Fig Functional subnetwork of the calcium homeostasis. *Functional subnetwork* of calcium homeostasis including APP and its family members APLP1 and APLP2. The color code corresponds the pie chart diagram. Of note, APP appears as a highly connected node within this network. Abbreviations are the respective gene names of individual proteins as given in UniProt database and in the supplementary information S1 Table.
(PDF)

S1 Text Information about the PPI network. Binary protein-protein interaction (PPI) data were extracted from protein interaction databases (February 2015). Here, additional information about PPI networks is provided.
(PDF)

S1 Cytoscape 3 File Visualizing, integrating, and analyzing networks. For visualizing, integrating, and analyzing networks, Cytoscape 3.2.1 was used to create the actual PPI network.
(CYS)
