# Supplementary figures and images for: APP Is a Context-Sensitive Regulator of the Hippocampal Presynaptic Active Zone
Source: PLoS Comput Biol. 2016 Apr 19;12(4):e1004832. doi: 10.1371/journal.pcbi.1004832 (PMC4836664; doi:10.1371/journal.pcbi.1004832)

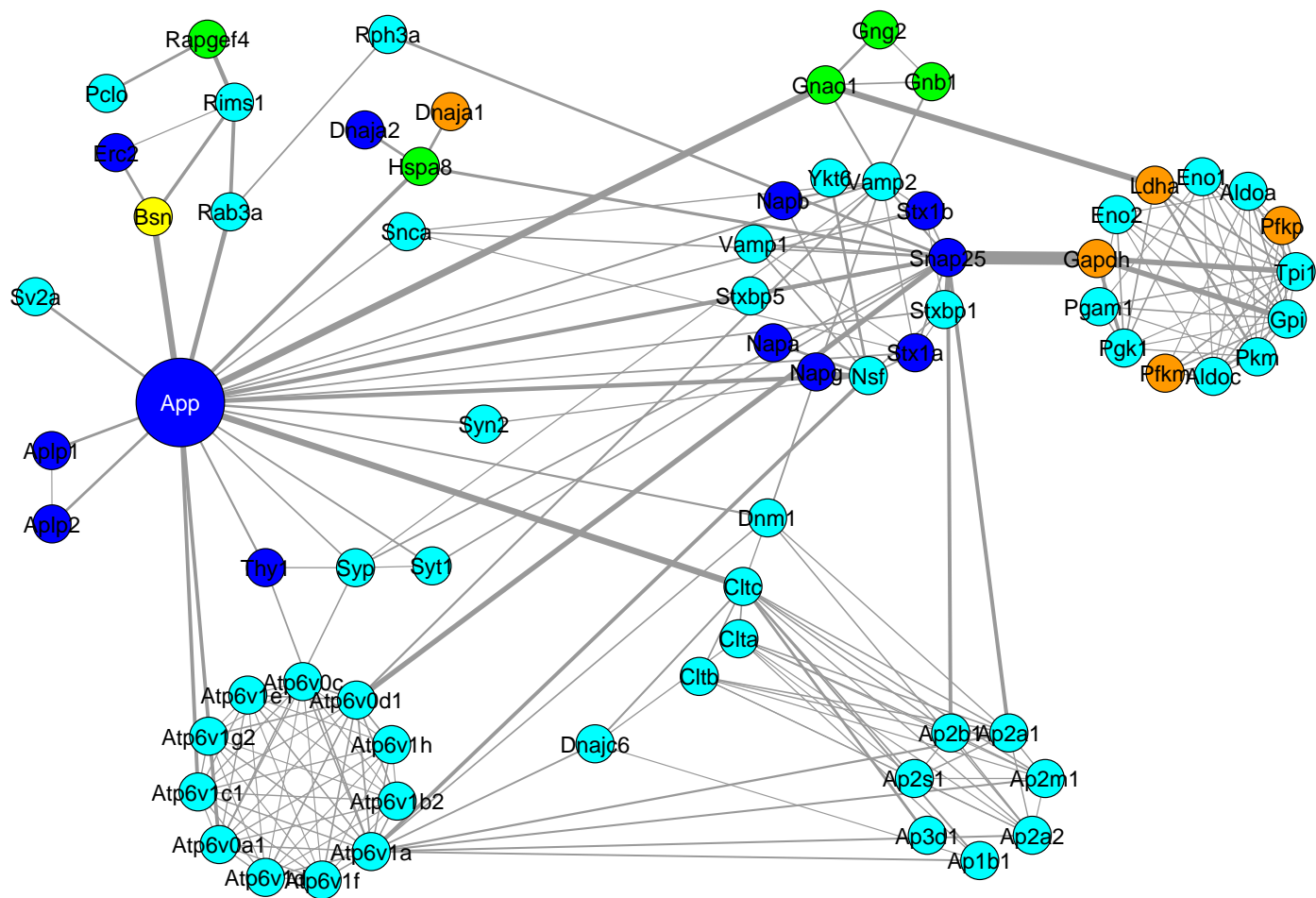

Supplement: S1 Fig — Functional subnetwork of the synaptic vesicle cycle including APP and its family members APLP1 and APLP2. The color code corresponds to the pie chart diagram. Of note, APP appears as a highly connected node within this network. Abbreviations are the respective gene names of individual proteins as given in UniProt database and in the supplementary information S1 Table. (PDF) [file pcbi.1004832.s002.pdf]

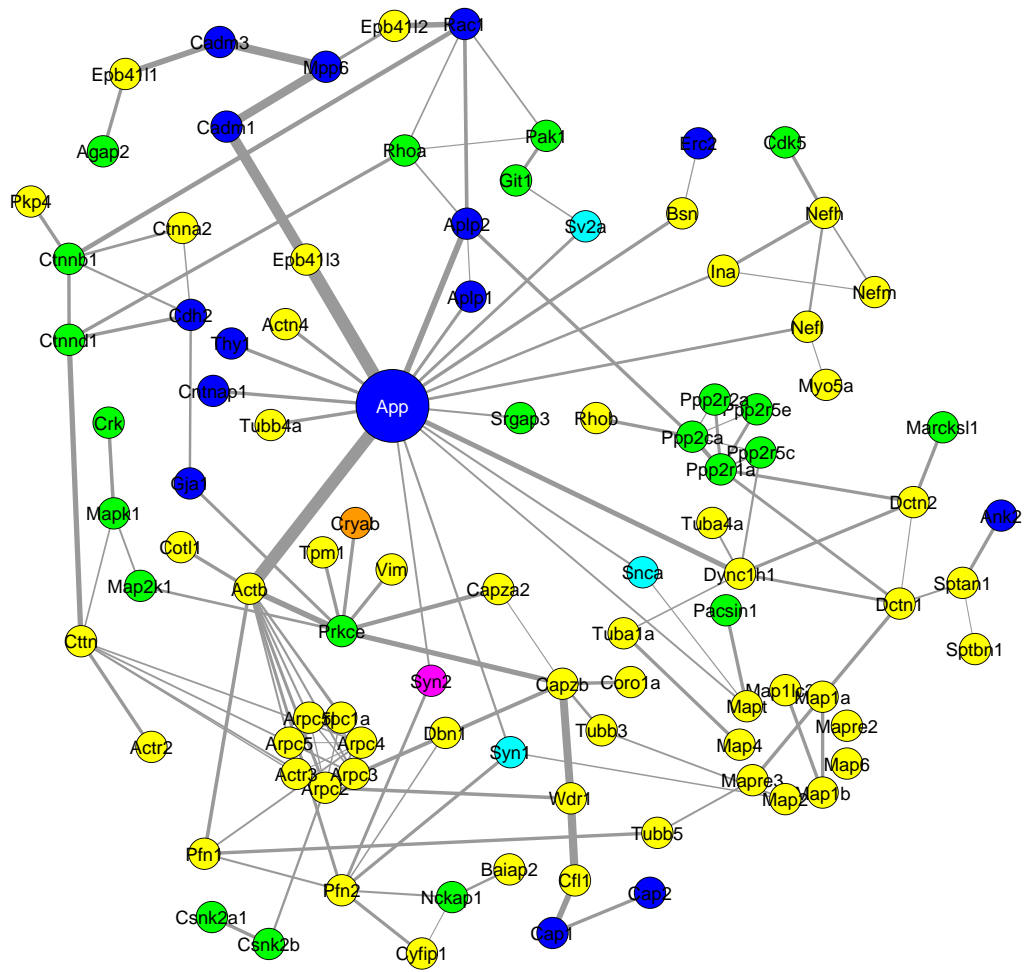

Supplement: S2 Fig — Functional subnetwork of cytoskeleton organization including APP and its family members APLP1 and APLP2. The color code corresponds to the pie chart diagram. Of note, APP appears as a highly connected node within this network. Abbreviations are the respective gene names of individual proteins as given in UniProt database and in the supplementary information S1 Table. (PDF) [file pcbi.1004832.s003.pdf]

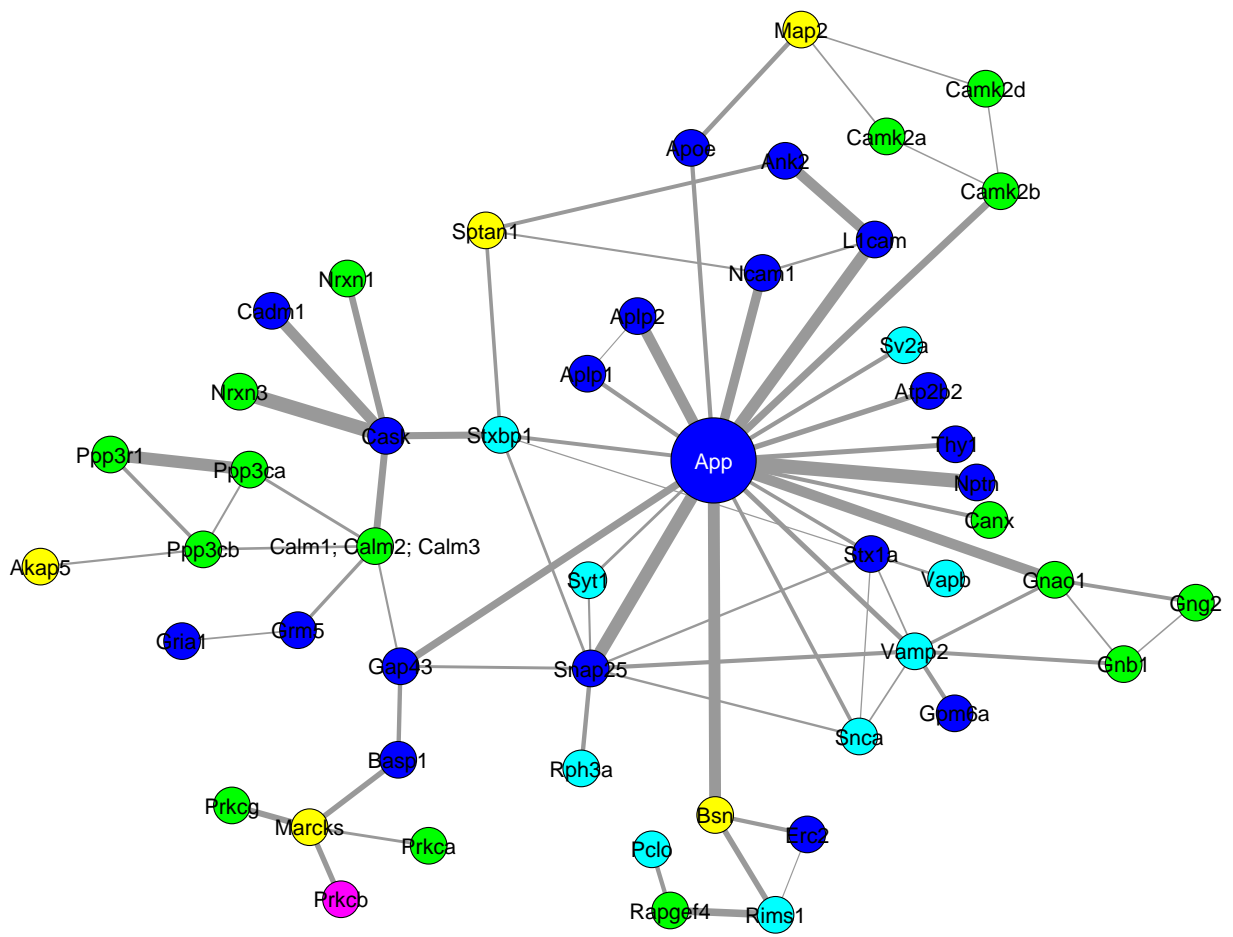

Supplement: S3 Fig — Functional subnetwork of calcium homeostasis including APP and its family members APLP1 and APLP2. The color code corresponds the pie chart diagram. Of note, APP appears as a highly connected node within this network. Abbreviations are the respective gene names of individual proteins as given in UniProt database and in the supplementary information S1 Table. (PDF) [file pcbi.1004832.s004.pdf]
