# Supplementary material for: APP Is a Context-Sensitive Regulator of the Hippocampal Presynaptic Active Zone
Source: PLoS Comput Biol. 2016 Apr 19;12(4):e1004832. doi: 10.1371/journal.pcbi.1004832 (PMC4836664; doi:10.1371/journal.pcbi.1004832)
Supplement: S1 Text — Binary protein-protein interaction (PPI) data were extracted from protein interaction databases (February 2015). Here, additional information about PPI networks is provided. (PDF) [file pcbi.1004832.s005.pdf]

Introduction to PPI networks

Proteins are the molecular machines of a cell. The whole set of proteins in a cell or a specific compartment of a cell is called proteome. Proteins enable the cell to fulfill its enormous variety of functions. Proteins build, amongst others, the cytoskeleton, molecular motors, receptors, transporters and more. Mostly, proteins do not work on their own, but either assemble into complexes with other proteins, or organize themselves in functional modules. Protein complexes, e.g., the SNARE complexes, are composed of several subunits and act as a unit. Functional modules are sets of proteins, which are involved in the same or similar biological processes, e.g., the cell cycle or the endocytosis, but do not necessarily interact at the same time and place. The assumption here is, that proteins involved in the same processes are more likely to act with each other than with proteins from different processes. Complexes and functional modules imply physical interactions between the proteins. Graph theoretical methods are applied to analyze these interactions. The results of these analyses aid in the understanding of cellular processes and their organization.

The physical interactions between the proteins are integrated into a network of protein-protein interactions (PPI). A PPI network is built of nodes and connections, where the nodes represent the proteins, and the connections between the nodes represent the possible physical interactions between the proteins. A PPI network represents a static model of all physical interactions reported for the proteome of interest. It does not integrate quantitative data as, e.g., the strength of the interactions, which domains interact, or spatiotemporal information. Rather, a PPI network is suited to analyze the organization of proteins or a proteome in terms of the functional organization.

Protein-complexes are represented as distinct graph structures, which are called cliques. A clique is a subgraph in which all noded are connected to each other, i.e., the subgraph is maximal connected (Fig. 1). This property makes putative molecular complexes detectable with algorithmic solutions (Bron and Kerbosch, 1973).

Detection of functional modules is commonly referred to as community detection. Community detection in PPI networks is an active area of research (Fortunato, 2010). Using the topology of a PPI network we can find densely connected parts and partition the network into meaningful subnetworks. Based on additional information as, e.g., the localization or functional annotations of the involved proteins, the subnetworks can be functional classified. The classification of a subnetwork adds another layer of information that is used to reveal the functional organization of the entire network.

A global analysis of network properties gives information about the topological organization of the network. One of the most prominent properties of biological networks is scale-freeness. In a scale-free network a few nodes are highly connected while most of the nodes only have a few connections (Barabási and Albert, 1999). In other words, the probability that a protein has k connections decreases with high k. The distribution of the probabilities for k connections is called the node degree distribution. For scale-free networks, this distribution follows a power-law and appears linear in a log-log plot. The concept of scale-freeness, therefore, is used to distinguish biological networks from random networks, which follow a Poisson distribution. The highly connected nodes are referred to as hubs and, based on their high connectivity, play an important role in a PPI network. The hub proteins have been shown to be essential for the organism in eukaryotic organisms (Jeong et al., 2001).

Identification of hub proteins is facilitated by the application of centrality analysis. Centralities are numerical values that are assigned to individual proteins in the network. Based on the centralities, the nodes are ranked and the most important ones are identified. In the case of

hub identification, the numerical values used as centralities are simply the number of interactions (Fig. 2 (A)). For example, APP has 71 connections in the PPI network of the PAZ and, therefore, is classified as a hub in the PPI network of the PAZ. Another centrality measure is the shortest path betweenness centrality. The betweenness centrality measures the ability of a protein to monitor communication between other proteins. Therefore, a protein with a high betweeness centrality has a linker or bridging function in the network. APP has a betweenness of ~0.12. The shortest path betweeness centrality utilizes the shortest paths between every node of the network. A protein is considered to be central if it participates in numerous shortest paths than other proteins (Fig. 2 (B)). Removing of high betweenness proteins from the network has been proposed for an optimal partition of the network (Girvan and Newman, 2002).

The clustering coefficient measures the degree of connectivity in the neighborhood of a protein in the network. A protein with a high cluster coefficient is part of a densely connected part of the network. In a dense part of a network, if protein A is connected to protein B, and protein B is connected to protein C, the probability is high that protein A is also connected to protein C (Fig. 2 (C)). The cluster coefficient can also be measured for the entire network and indicates the average cluster coefficient of all proteins in the network.

The combination of different centrality measures enables a deeper understanding of the role of a protein in a network. A protein might be important for the structure for a network even if it is lowly connected. In this case a high betweenness centrality value might suggest a bridging role for the protein.

References:

Barabási, Albert-László, and Réka Albert. *Emergence of scaling in random networks.* Science 286.5439 (1999): 509-512.

Girvan, Michelle, and Mark EJ Newman. *Community structure in social and biological networks.* Proceedings of the National Academy of Sciences 99.12 (2002): 7821-7826.

Hahn, Matthew W., and Andrew D. Kern. *Comparative genomics of centrality and essentiality in three eukaryotic protein-interaction networks.* Molecular Biology and Evolution 22.4 (2005): 803-806.

Jeong, Hawoong, et al. *Lethality and centrality in protein networks.* Nature 411.6833 (2001): 41-42.

Bron, Coen, and Joep Kerbosch. *Algorithm 457: finding all cliques of an undirected graph*. Communications of the ACM 16.9 (1973): 575-577.

Fortunato, Santo. *Community detection in graphs.* Physics Reports 486.3 (2010): 75-174.

Figure 1:

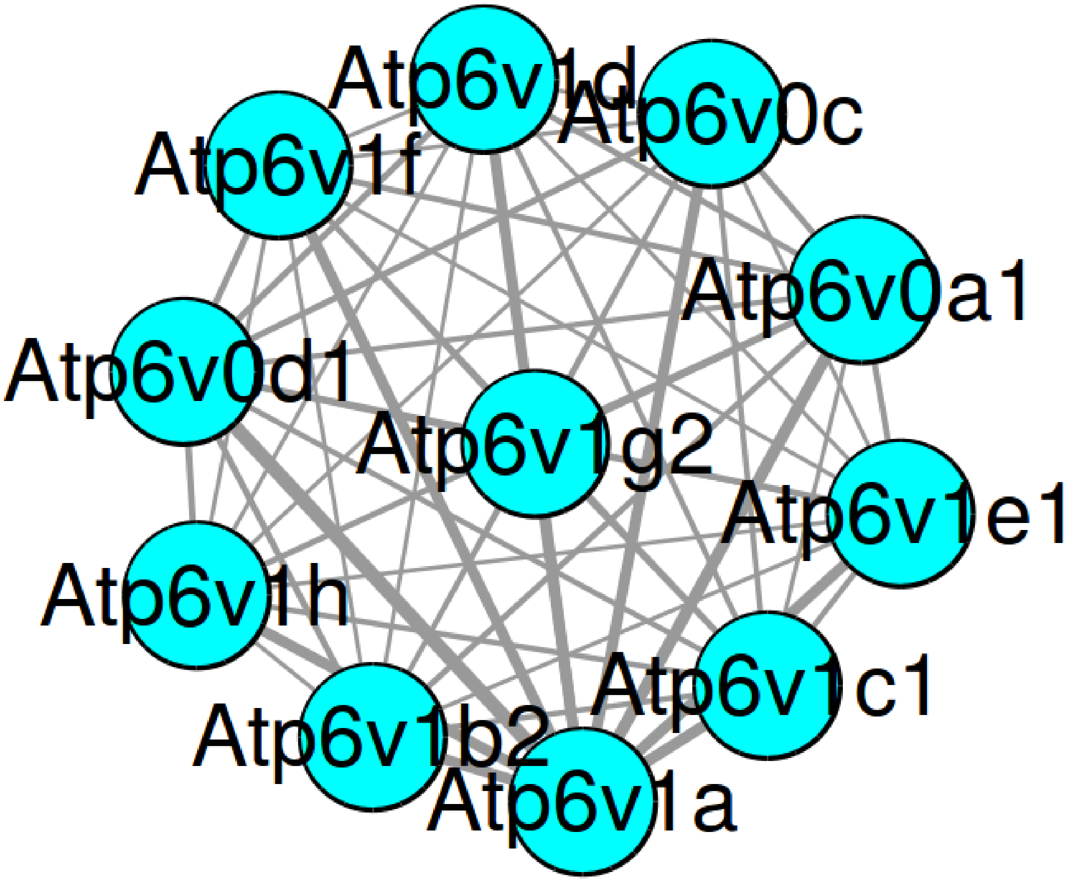

Figure 1: vATPase protein-complexes represented as a clique. A clique is a subgraph in which all noded are connected to each other, i.e., the subgraph is maximal connected.

Figure 2:

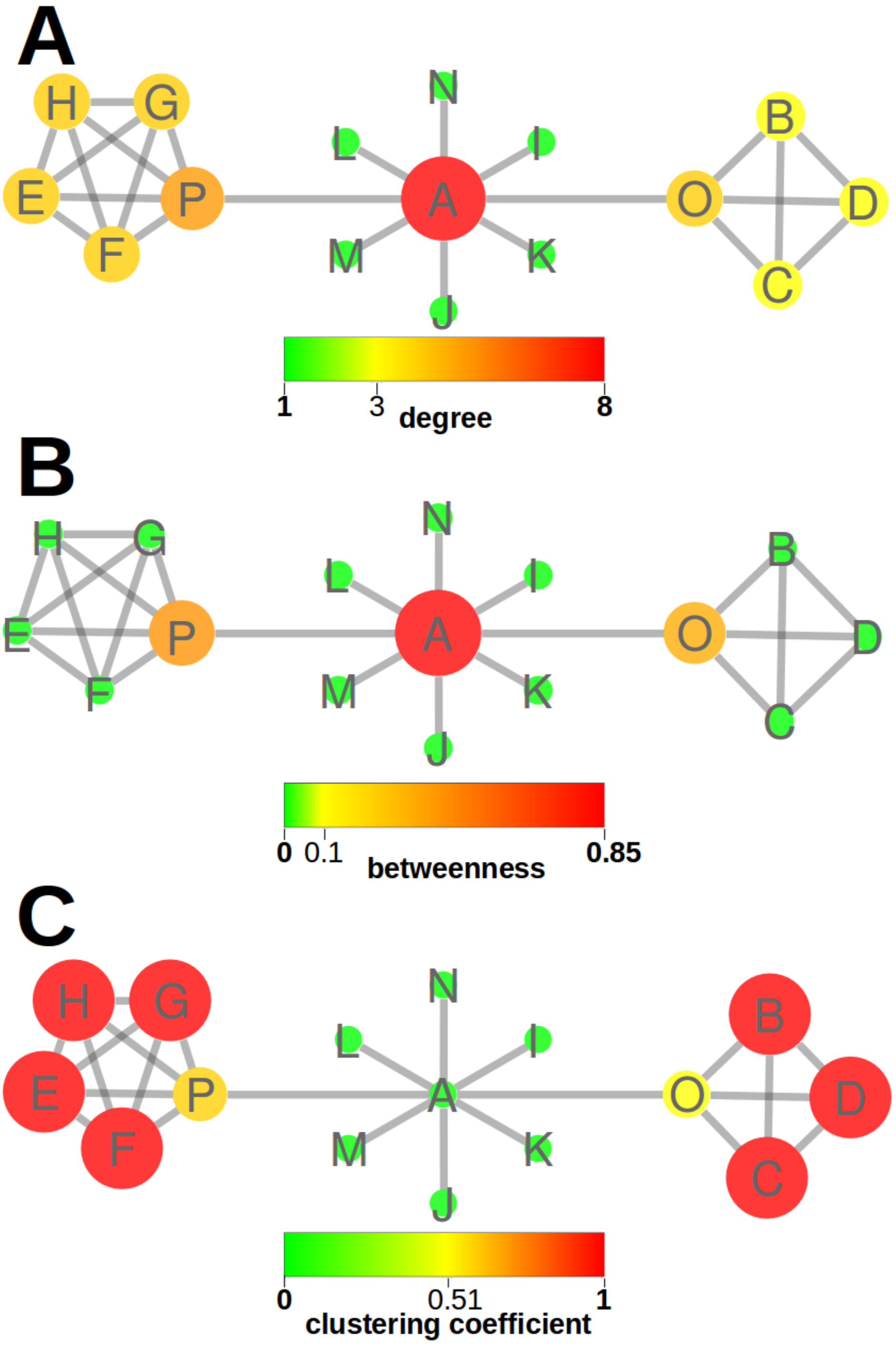

Figure 2: Centrality analysis: For hub identification, the numerical values used as centralities are simply the number of interactions (A). A protein is considered to be central if it participates in numerous shortest paths than other proteins (B). The clustering coefficient measures the degree of connectivity in the neighborhood of a protein in the network. A protein with a high cluster coefficient is part of a densely connected part of the network (C).
